# Encapsulated salts in velvet worm slime drive its hardening

**DOI:** 10.1038/s41598-022-23523-z

**Published:** 2022-11-10

**Authors:** Yendry Regina Corrales-Ureña, Fabienne Schwab, Efraín Ochoa-Martínez, Miguel Benavides-Acevedo, José Vega-Baudrit, Reinaldo Pereira, Klaus Rischka, Paul-Ludwig Michael Noeske, Alexander Gogos, Dimitri Vanhecke, Barbara Rothen-Rutishauser, Alke Petri-Fink

**Affiliations:** 1 Adolphe Merkle Institute, University of Fribourg, Chemin Des Verdiers 4, 1700 Fribourg, Switzerland; 2 Faculty of Production Engineering, University of Bremen, Am Fallturm 1, 28359 Bremen, Germany; 3 National Laboratory of Nanotechnology LANOTEC - National Center of High Technology CeNAT, 1.3 Km North of the United States Embassy, San José, Costa Rica; 4 School of Chemistry, National University, Heredia, Costa Rica; 5 Adhesive Bonding Technology and Surfaces, Fraunhofer Institute for Manufacturing Technology and Advanced Materials IFAM, Wiener Straße 12, 28359 Bremen, Germany; 6 EMPA, Swiss Federal Laboratories for Materials Science and Technology, Lerchenfeldstrasse 5, CH-9014 St. Gallen, Switzerland; 7 Department of Chemistry, University of Fribourg, Chemin du Musée 9, CH-1700 Fribourg, Switzerland

**Keywords:** Biophysical chemistry, Nanoscale biophysics

## Abstract

Slime expelled by velvet worms entraps prey insects within seconds in a hardened biopolymer network that matches the mechanical strength of industrial polymers. While the mechanic stimuli-responsive nature and building blocks of the polymerization are known, it is still unclear how the velvet worms’ slime hardens so fast. Here, we investigated the slime for the first time, not only after, but also before expulsion. Further, we investigated the slime’s micro- and nanostructures in-depth. Besides the previously reported protein nanoglobules, carbohydrates, and lipids, we discovered abundant encapsulated phosphate and carbonate salts. We also detected CO_2_ bubbles during the hardening of the slime. These findings, along with further observations, suggest that the encapsulated salts in expelled slime rapidly dissolve and neutralize in a baking-powder-like reaction, which seems to accelerate the drying of the slime. The proteins’ conformation and aggregation are thus influenced by shear stress and the salts’ neutralization reaction, increasing the slime’s pH and ionic strength. These insights into the drying process of the velvet worm’s slime demonstrate how naturally evolved polymerizations can unwind in seconds, and could inspire new polymers that are stimuli-responsive or fast-drying under ambient conditions.

## Introduction

Biomedical adhesives are challenged by the need for significant adhesion to soft tissues, even in wet environments^1^. Knowledge-based material development can gain inspiration from biological processes and materials from natural sources that allow for tailoring adhesion and, thus, show a potential not only for biomedical but also for technological breakthroughs, e.g. in 3D bioprinting of biomacromolecules applications^2,3^. In this view, the unique slime secretion used by velvet worms—invertebrates from the phylum Onychophora—for defense, prey capture and parental feeding^4,5^ is exemplary. The velvet worm *Epiperipatus biolleyi,* which is found in Central America^6^, produces a white sticky slime that strongly adheres to a wide variety of materials such as wood, metals, glass, and biological tissues.

The slime-expelling mechanism of velvet worms has been investigated previously using anatomical images and high-speed videography^7^. The worms possess a syringe–hose system in which the muscles surrounding the slime reservoir are contracted, forcing it through a funnel-shaped duct, at which the fluid velocity is increased. The slime then passes through two oral papillae (*i*.*e*. elastic oral tubes) that oscillate at a frequency of ~ 30 Hz, producing two slime jets that form a sticky fiber mesh lined with micrometer-sized liquid droplets. While this process is well known, and the expelled slime has been partially characterized^8–16^, the physicochemical processes that drive the rapid drying of the slime after expulsion and mechanical stimulation are only partially known. The expelled slime remains a viscous liquid after expulsion, and upon mechanical stimuli, for example, due to the movements of an entrapped insect, it transforms within seconds to a highly expandable rubber-like and then stiff glassy material^8,9^. This liquid–solid transition can occur as fast as within 10 s to 1 min after expulsion and mechanical stimulation^8^.

An initial study on the slime of *Euperipatoides rowelli* proposes that velvet worm slime consists of protein nanoglobules, which self-assemble into microfibrils after the application of shear forces^9^. Baer et al. showed that these fibrils are formed by highly phosphorylated proteins that have a *β*-crystallite structure in the storage phase^14,15^. Recently, Lu et al. reported that the most abundant proteins, ES_P1 and ES_P2, form multi-protein complexes and are mostly in random coil conformation in the soluble state. The N-terminus are enriched in Gly and Ser, and they suggested that proteins with this terminus could be responsible for the liquid–liquid phase separation^16^. Bauer et al. suggested that these proteins forming the *β*-crystallites suffer conformational changes, or partial protein unfolding, during the stretching produced by the shear forces. When dried, the proteins lose mobility and stay in this conformation, but when rehydrated, they gain mobility, so the nanoglobules are formed again, suggesting that the nanoglobule-to-fiber formation is reversible^14,17^.

Our previous results showed that the fiber formation process was reversible after dissolving the slime in the same amount of water as the native slime (several hours are necessary) or acetic acid-diluted solutions (almost immediately)^8^. However, the fast liquid-to-solid transition of the bulk slime cannot be reproduced; the water-reconstituted slime is more elastic and loses its glassy appearance. We reported the presence of nanoglobules in our recent work on solidified expelled slime from a different species (*Epiperipatus hilkae*) that had not yet experienced an external mechanical stimulus. We also discovered fibers, crystalline domains, and abundant electron-dense micro- and nanostructures with higher metal contents compared to the nanoglobules, and the lipids previously reported^8^. The electron-dense particles could not be reversibly assembled from the reconstituted hardened slime as it was previously done with the nanoglobules. Due to their abundance, we hypothesized that these electron-dense structures could play a vital role in the slime’s rapid liquid-to-solid transition.

In the present work, we performed an in-depth physicochemical characterization of velvet worm slime’s electron-dense particles and investigated changes in their structure and the properties of the slime. We characterized both the expelled slime (before and after applying external mechanical stimulus) and unexpelled slime (extracted in situ from the reservoir). To rule out artifacts due to contaminations (onychophorans have an open circulatory system^18^), we also analyzed the hemolymph and nano- and microparticles isolated thereof. The slime and nano- and microparticles were analyzed by cryo-transmission electron microscopy (cryo-TEM), selected area electron diffraction (SAED), scanning transmission electron microscopy (STEM), Energy-dispersive X-ray spectroscopy (EDS), polarized microscopy, Attenuated Fourier-transform Infrared Spectroscopy (ATR-FTIR) and protein and lipid staining. The chemical composition of the slime and hemolymph was compared using EDS and Inductively Coupled Plasma Spectroscopy (ICP). The influence of the salts on the tension pull-off adhesion force between two glass blocks and Young’s modulus of the slime was determined by mechanical dynamical analysis (DMTA) and atomic force microscopy (AFM). Therefore, the influence of the salts and small molecular weight compounds in the Storage G′ and Loss modulus G″ of the slime reconstituted in water at a concentration of 40 mg/mL was analyzed.

## Results

### Characterization of the bulk slime and hemolymph

**Figure 1:**
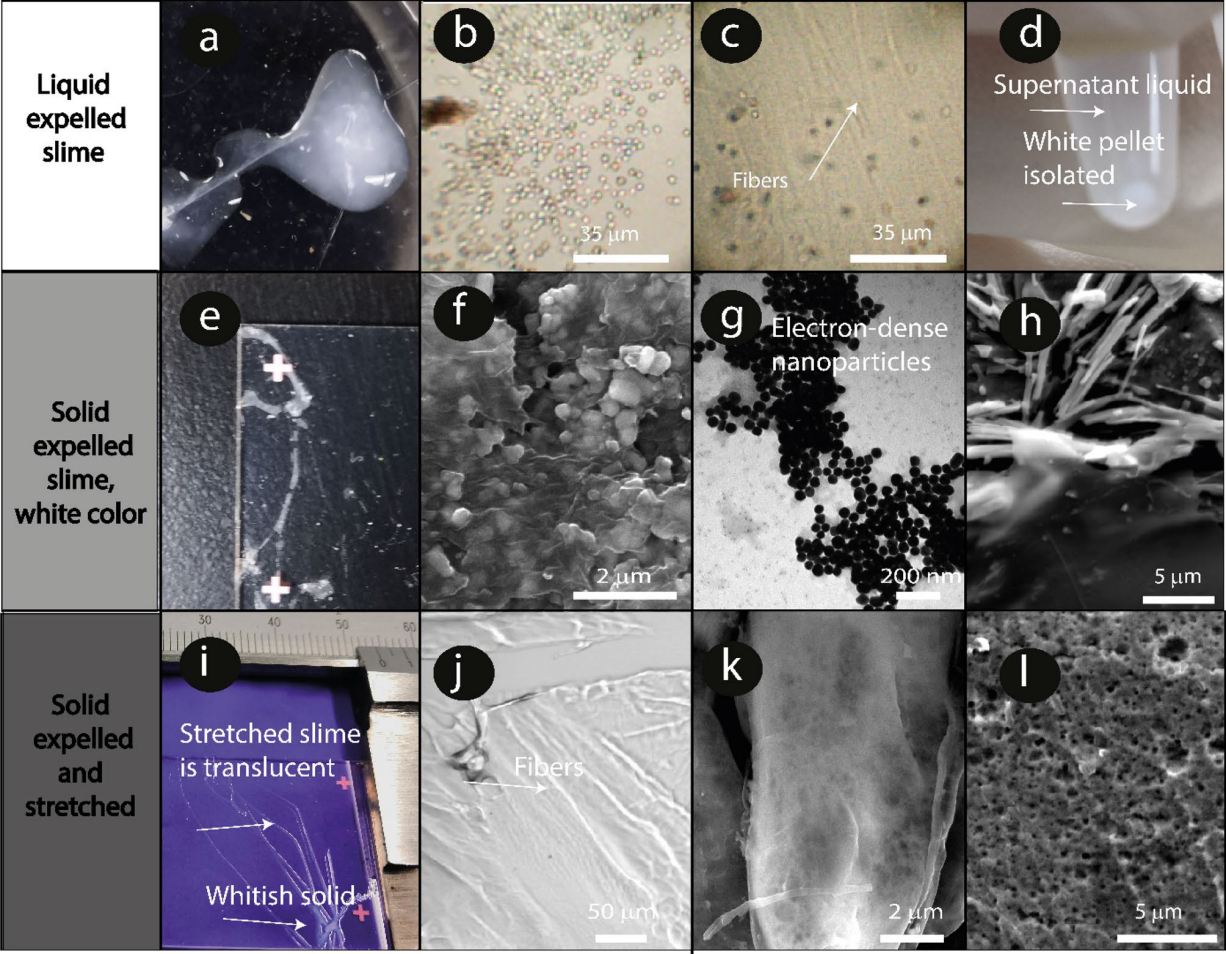
(**a-d**): Liquid expelled slime, (**e–h**): solid expelled slime, (**i-l**) solid expelled slime after a mechanical stimulus (stretching). (**a)** Photograph of the white expelled slime on a polystyrene Petri dish. Note that the slime remains liquid without external mechanical stimuli such as stretching. The sample was handled carefully to avoid shear stress. (**b)** Bright-field image of the dispersed roundish-microparticles in the liquid protein slime matrix. (**c**) Bright-field image of the fibers and microparticles dispersed in the liquid slime. (**d)** White pellet precipitated from the viscous white slime solution. Note that the liquid supernatant is translucent. (**e**) Photograph of the dried expelled slime that did not experience any external mechanical stimulus on a glass slide. Note that this slime remained white. (**f**) SEM micrograph of nano- and microparticles dispersed in the slime. (**g**)TEM image of electron-dense roundish nanoparticles < 500 nm size. **(h**) SEM image of the fibers and microparticles in the solid expelled slime. (**i**) Expelled slime on a glass slide. Note that the slime in the upper part of the image is translucent (it was stretched) and that the unstretched slime in the lower part remains white. (**j**)  Fibers observed in the translucent area (**k**) SEM image of the slime threads formed. Note the absence of nano- and microparticles. (**l**) SEM cross-section image of one slime thread. A porous structure is present.

We collected slime expelled by velvet worms and characterized it optically and with high-resolution microscopy. Figure 1 compares the liquid expelled slime and the solid slime before and after an external mechanical stimulus.

The liquid expelled slime was white and with no glassy appearance (Fig. 1a, Supplementary Video 1), and hundreds of nanometers particles and fibers were dispersed in the liquid phase before it experienced an external mechanical stimulus (Fig. 1b,c). A white pellet containing micro and nanoparticles was obtained when the slime was allowed to flow slowly into an Eppendorf tube and then centrifugated, Fig. 1d. The slime’s white color and the supernatant’s color after that centrifugation suggest that the slime is an emulsion. The remaining supernatant solution was translucent. The environmentally dried expelled slime is a white solid (Fig. 1e) and contains electron-dense nano and microparticles dispersed in the protein matrix, Fig. 1f,g. Preformed microfibers were also observed, Fig. 1h. The size of the electron-dense particles was similar for each slime sample but differed from one specimen to another. The electron-dense core areas of the particles varied from 50 nm to 3 µm. Figure 1g shows the slime containing the smallest electron-dense particles found among the samples analyzed, confirming that the darkest color is associated with the presence of heavier elements, e.g. carbon, nitrogen, and oxygen and not the particle thickness.

Some worms were dissected to determine if these electron-dense microparticles were also present in the stored slime and not just external contamination. Using a CO_2_ chamber as the first dissection step, make the specimen calm asleep without slime expulsion before the next dissection steps, which were: introducing them into a chloroform vapor chamber, removing the body impurities using a water bath, opening the dorsal area, and removing the hemolymph surrounding the reservoir, as the velvet worms have an open circulatory system^18–21^. Finally, the reservoir was separated and opened by small scissors as fast as possible to collect the slime (Supplementary Fig. 1).

The slime extracted directly from the reservoir showed similar features compared to the expelled slime that didn't experience an external mechanical stimulus: white color, presence of dispersed micro and nanoparticles in suspension, and fibers (Supplementary Fig. 1). These results suggested that the crystalline microparticles are not formed as an artifact of the drying, during the slime expulsion or external contamination. Furthermore, the pellet precipitated from 100 µL was clearly visible at the Eppendorf bottom. The approximate weight of the pellet isolated ranged between 12–6 wt% of the initial mass. As described in the following sections, the cryo-TEM analysis confirms the presence of crystalline nanoparticles and microparticles. To verify that these microparticles and nanoparticles were not vesicles released in hemolymph from apoptotic cells or released by cells in a process that could be similar a the release of polyphosphate for platelets during coagulation^22^, hemolymph from the dorsal area was analyzed and compared with the slime. Therefore, the presence of cells in the expelled slime was also investigated.

The elemental composition of the expelled slime and the hemolymph obtained from the dorsal area were analyzed by ICP and EDS to determine if the vesicles could be derived from the hemolymph. Both fluids present similar elemental compositions in terms of Na, O, P, N, Ca, Mg, K and Al, but at different concentrations; Na, P, Ca and K are higher metal concentrations for both fluids (Supplementary Fig. 2, Supplementary Table 1). Even though the elemental composition was similar, the electron-dense particles could not be found in the dried hemolymph fluid containing a high amount of cells. Dried hemolymph showed mainly cells, NaCl crystals at the border of the dried fluid, calcium oxygen-based particles with no defined preferential morphology, and roundish micrometer structures with a lower amount of P, Na and O than the crystalline particles (Supplementary Fig. 2). Furthermore, the slime is acellular, as shown by the paraffin negative eosin/hematoxylin staining cross-sections of the paraformaldehyde cross-linked solid slime (Supplementary Fig. 3) and also containing glycans. Masson staining and collagen immunostaining of the expelled slime suggests the presence of collagen fibers dispersed in the slime. Collagen fibers are located between the reservoir muscles and lumen (Supplementary. Fig. 4). Therefore, we cannot discard the mixture with a certain hemolymph amount during the expelling process. Still, the hemolymph is not likely to be the source of the dispersed electron-dense particles, as we did find this type of nanoparticle even in the concentrate suspension as described above. Furthermore, the slime extracted from inside the reservoir after removing the hemolymph from the surrounding organs is white and presents similar electron-dense particles (Supplementary Fig. 1).

**Figure 2:**
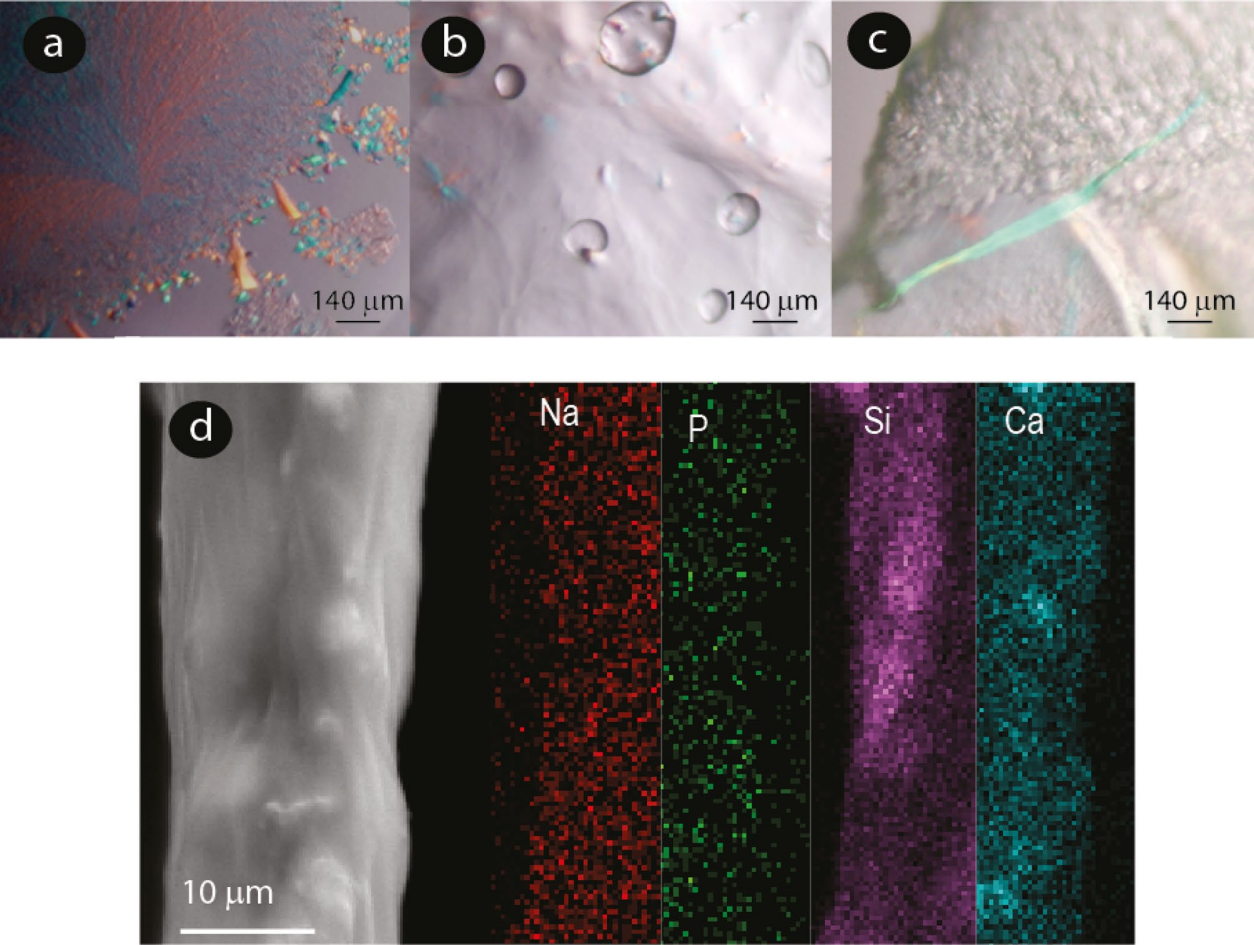
Microscopy images of slime. (**a**) Polarized microscopy images of slime expelled before experiencing an external mechanical stimulus showing crystalline structures. (**b**) and (**c**) slime expelled after an external mechanical stimulus. (**d**) SEM micrograph of an expelled slime fiber and elemental mapping using EDS.

The extracted from the reservoir and expelled slime protein matrix before an external mechanical stimulus formed lamellar layers in which preformed fibers appear preferentially aligned (Supplementary Figs. 5,6). The slime birefringence shows that the slime is stored as a liquid crystalline fluid in the reservoir, Fig. 2a. A slime matrix consisting of proteins forming *β*-crystallites in the storage phase has been suggested by Bauer et al.^15^, who described interlattice spacings, seem to be consistent with those of the electron-transparent few nm-sized crystallites that we found in the protein matrix. These observations are in line with previously reported birefringence of the fibers and particles composing the expelled slime of *Epiperipatus hilkae*^8^. Liquid crystalline ordering has also been suggested as a possible storage condition for silks in other animals, such as spiders^23,24^, and can occur in a wide variety of other biological molecules, such as DNA, collagen, and cellulose. This fluid state may help to decrease the flow viscosity and, consequently, the energetic cost of its secretion^25^. Harrington et al. suggested that the slime protein changes from a *β*-crystallite state to a random coil state when stretched^17^. When the worm expelled the slime all over a glass slide, it formed transparent threads, as seen in Fig. 1i, upper part. The bottom part, where the liquid slime settled without being stretched, remained white. The transparent threads expelled on a glass slide by the worm show an inner crystalline area surrounded by an amorphous coating (Supplementary Fig. 7a), suggesting a phase separation that started during the expelling process. Micrometric fibers were observed but not microparticles in the transparent areas, Fig. 1j,k. Figure 1h,j show how the bulk material did not exhibit a high amount of particles on the surface or in the inner area. Figure 2b,c shows the loss of crystalline order after applying an external mechanical stimulus to the slime. Only randomly dispersed micron-sized fibers that polarized the light were observed. After experiencing an external mechanical stimulus, the expelled slime had a porous structure that could indicate a discontinuous protein network formation or phase separation due to the mechanical stimulus and CO_2_ formation (Fig. 1l and supplementary Fig. 7a)^26,27^. This structure could facilitate water diffusion within/through the fibers and their interface with air. The mechanism and role of this porous network formation remain to be fully understood.

When both dried slime samples, i.e., the one expelled and the one extracted from the reservoir, were rehydrated with the approximate amount of water of the liquid slime, they became transparent after mixing with water (80 wt% water), and elastic-like threads could be spun from the suspensions, but no fast-drying took place in this case (Supplementary video 2).

A comparison of the FTIR spectra of the expelled slime (remaining white) before and after experiencing external mechanical stimulus (transparent) shows a shift in the amide I and amide II bands from 1625 to 1634 cm^−1^ and from 1533 to 1515 cm^−1^ (Supplementary Fig. 8a). This shift can be correlated to a change in the protein assembly and interactions with the chemical species surrounding the proteins. The band at 1625 cm^−1^ indicates the presence of *β*-sheets in the expelled slime without external mechanical stimulus^28^. A pronounced peak at 1723 cm^−1^ could be associated with the concentration of the lipidic fraction surrounding the fibers (FTIR signal is obtained from the outer layers, 2 µm depth). This lipidic coating surrounding the fibers could also help to expel the water from the first contact surface, increase the compatibility with insects' cuticles and protect from early disaggregation^29,30^.

Dynamic scanning analysis of the expelled slime (Supplementary Fig. 8b), in a range below its degradation temperature (Supplementary Fig. 8c), showed an endothermic event at 53,6 °C that could be correlated to protein active water loss and conformational changes. Lu et al.^22^ showed that the slime denatured, losing its fiber-forming formation properties at 70 °C^16^. This event was not detected in the expelled slime that experienced mechanical stimulus, which suggests conformational changes that could enhance the protein–protein interaction and change the mechanical properties^31^. The crystallization and melting of the lipids in the slime at 89–93 °C. Similar values have been obtained for waxes and lipids extracted from plants and insects^32^. The higher melting temperature could suggest that the lipids could form solid lipidic particles.

pH value of the expelled slime ranged from 5.0 to 5.5, while that of a transparent solution of rehydrated slime ranged from 6.5 to 7.0. There is a pH increase due to the dissolution of a basic component in the slime, which we report here to be the electron-dense particles shown in Fig. 1, as we will describe in the next sections. We then tested the stability of the dried films in both a neutral PBS buffer and a slightly acidic aqueous solution. Slime was partially stable in the neutral buffer (pH 7.4) but quickly dissolved in acidic media (pH 4.5, Supplementary Fig. 7b,c), suggesting the disassembly of the protein matrix composing the slime at low pH.

### Characterization of the electron-dense particles

**Figure 3:**
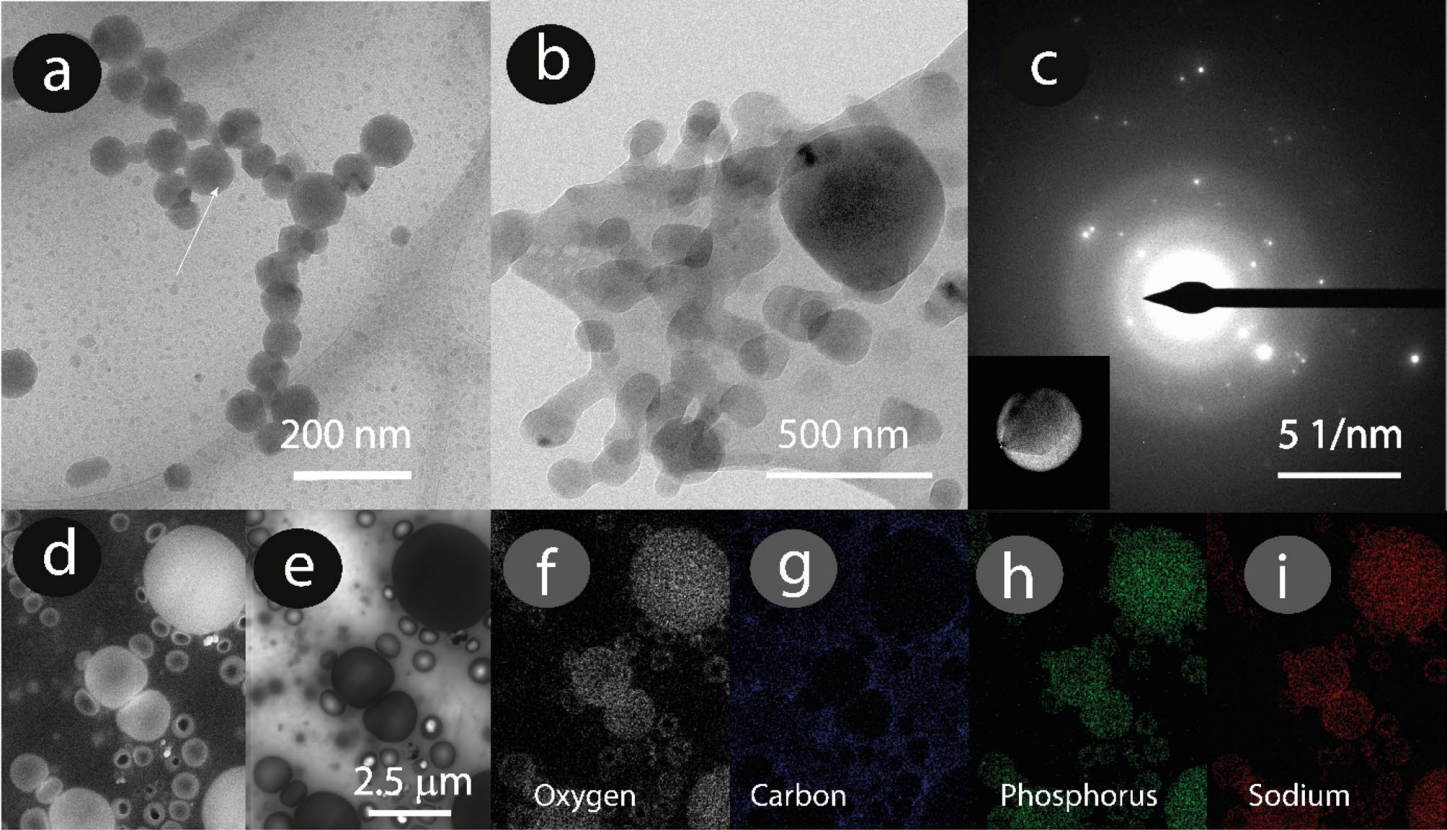
Electron microscopy of the slime’s electron-dense particles. (**a**) Cryo-TEM micrographs of the slime unexpelled and (**b**) expelled before experiencing an external mechanical stimulus, respectively. (**c**) Selected area electron diffraction (SAED) analysis of an electron-dense particle in the expelled slime. (**d-i**) STEM micrographs. (**d**) HAADF detector. (**e**) Bright-field detector. (**f-i**) Oxygen, carbon, phosphorus and sodium maps, respectively. Note that the P, Na and O are mainly detected in the electron-dense particles and not in the protein surrounding matrix.

The presence of electron-dense roundish particles was confirmed to be dispersed in the samples taken from the white freshly liquid unexpelled and expelled slime. Figure 3 a and b show the cryo-TEM micrographs of rounded and angular crystalline particles of 50 to 750 nm in diameter dispersed in the unexpelled slime and expelled before experiencing an external mechanical stimulus. The particles were sometimes freestanding, but more often, an electron-transparent coating interconnected several hundreds of these particles in a spring-like arrangement.

**Figure 4:**
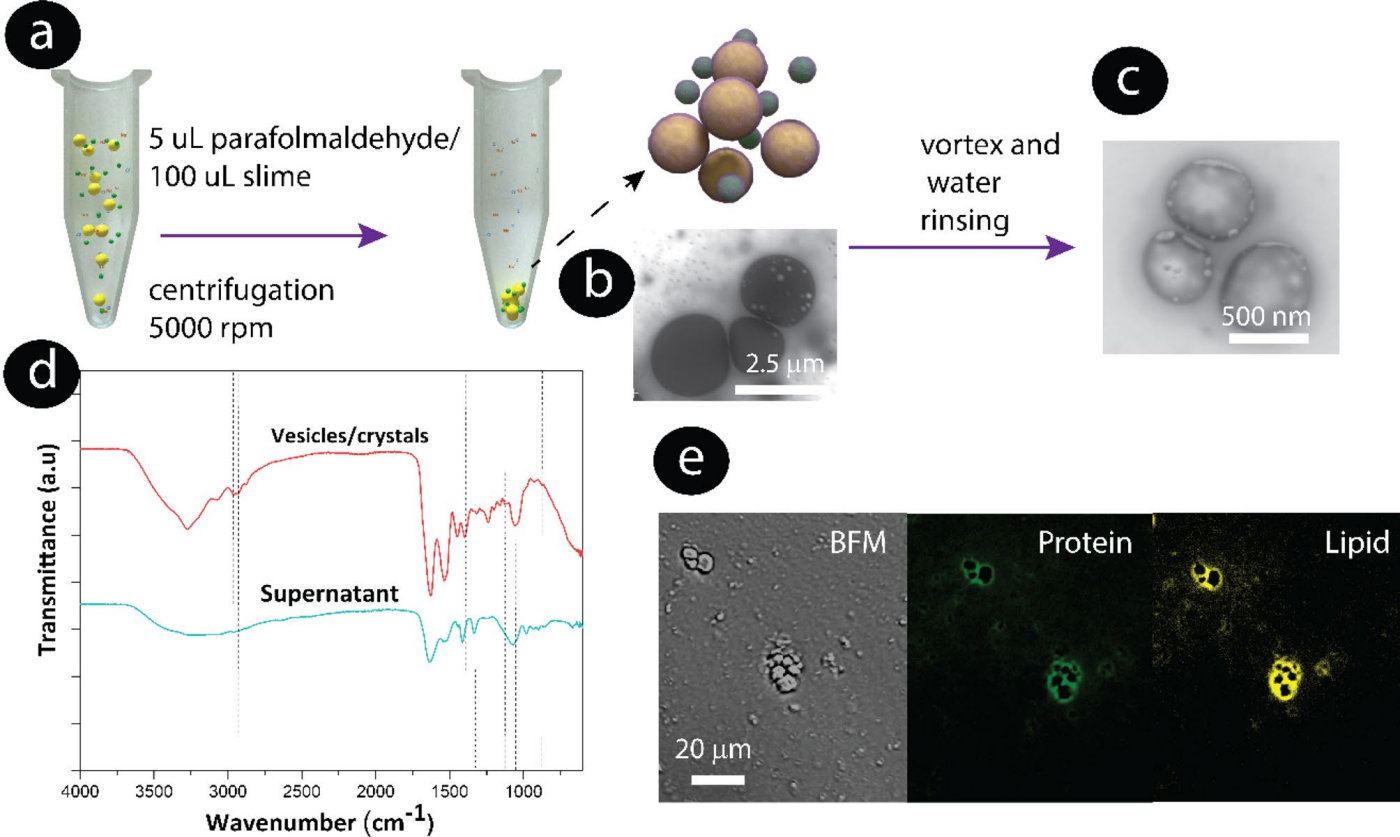
Particle and vesicle isolation process. (**a**) Scheme of the aldehyde fixation used to prevent the highly fragile carbonate and phosphate particles from reacting with each other. Centrifugation was used to collect the particle fraction of the slime. TEM image of (**b**) vesicles before rinsing and vortex. (**c**) Vesicles after rinsing with water and vortex. Note the morphology of the protein/lipid membrane left after rinsing the particles. (**d**) FTIR spectra of the pristine centrifuged pellet containing crystalline particles, protein fibers (e.g collagen), and supernatant. (**e**) Staining of particles isolated in the aqueous solution. BFM: Bright field image of particles isolated in the aqueous solution, focusing at the middle particle heigth, and after protein (green) and lipid (yellow). staining Note that the protein and lipid fluorescence signal exclusively highlights the outer layer and that the particle centers are dark.

The pellet isolated from the expelled slime (remaining white) using centrifugation was analyzed to determine the chemical composition of the crystals by different electron microscopy techniques. Before centrifugation, we fixed the particles with paraformaldehyde to stabilize them, as the shell most probably contained proteins, and the solubility and chemical composition was analyzed. Particles with diameters between 500 nm–3 µm were isolated from the expelled slime (Fig. 4a and Supplementary Fig. 9) at 5000 rpm. The centrifugation speed was optimized to decrease the precipitation of other globular protein structures found that could be correlated to protein aggregates, coacervates, or empty shells (Fig. 4b,c, Supplementary Fig. 10). The electron-dense particles were surrounded by a shell with different d-spacing than the electron-dense inner material (Supplementary Fig. 11). In general, the electron-dense particles were crystalline (Fig. 3c) with an electron-transparent nm-thick coating. Figure 3d–i are proof that the electron-dense particles encapsulate a higher amount of Na, O, and P than the surrounding areas.

The crystalline particles, cross-linked or not cross-linked, could not be completely dissolved in ethanol or chloroform as would be expected for lipids. But they were unstable and rapidly dissolved in water (Supplementary Figs. 12, 13). Inorganic salts such as carbonates and phosphates are poorly soluble in these solvents, but they do dissolve in water. When the particles were rinsed in water and vortexed, they left the cross-linked lipid-protein shells behind (Fig. 4c). Sodium or phosphorous was not detected (Supplementary Fig. 13).

The FTIR spectra of isolated particles from the expelled slime, Fig. 4d, showed two intense absorption bands at 1631 cm^−1^ and 1529 cm^−1^, corresponding to the amide vibrational bands I and II of proteins and peptides^33^. Furthermore, the band observed at 1400 cm^−1^ could be associated with carbonates^34^. Phosphates exhibit absorption bands at 1103 cm^−1^^35,36^ and silicates at approximately 1016 cm^−1^^37^. The bands at 2900 and 2990 a cm^−1^ are associated with C–C and C–H from lipids^8^. These findings suggest that the pellet contained proteins and/or lipoproteins. Protein and lipid staining of the pellet revealed that the micrometer-sized particles are not protein-based but rather stabilized by a lipid-protein coating, as we can observe in the hollow areas that were not stained inside the particles, as it was expected for protein particles (Fig. 4e). Jehle et al. showed that nitrogen was detected in protein composite particles of hundreds of nanometers within secretory vesicles mussel byssus cuticle using STEM-EDS opposite to the velvet worm vesicles that encapsulate mainly inorganic salts^38^. We speculate that this lipid-protein coating could serve for lubrication and help to isolate the particles to avoid the early reaction of inorganic cargo with the outer acidic media. To determine the presence of another type of particle, we also analyzed the supernatant solution. TEM grids of the deposited supernatant solution were analyzed before and after staining with osmium tetroxide and uranyl acetate. Supplementary Fig. 14 shows that lipid particles (without a electron-dense core) and protein aggregates that did not precipitate remained in the supernatant.

### Detail characterization of the isolated crystalline particles

**Figure 5:**
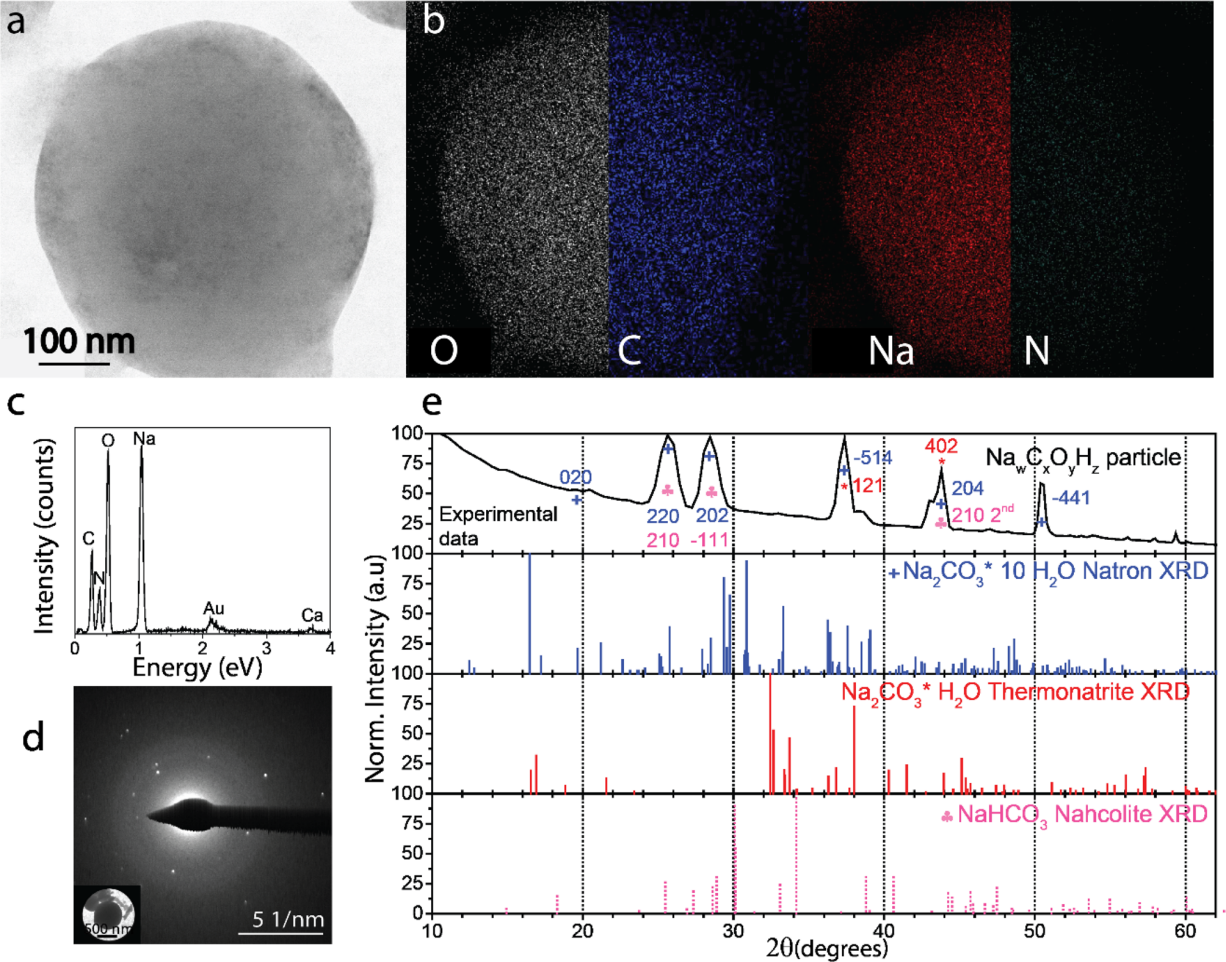
Morphology and chemical composition of isolated, dried carbonate particles in the expelled slime. (**a**) Typical rounded carbonate particle (brightfield STEM). (**b**) Elemental mapping of the particle shown in a. (**c**) SEM energy-dispersive X-ray spectrum of carbonate particle shown in (**d**). Elemental mappings of less abundant elements are presented in Supplementary Fig. 16. (**d**) TEM selected area electron diffraction (SAED) pattern of carbonate particle shown in the insert. (**e**) Crystalline phase analysis based on chemical composition and d-spacings. Note that the reference spectra were calculated from literature X-ray diffraction data of polycrystalline powders. In contrast, the present measurements show the electron diffraction from mostly single crystals for which the peak intensities can differ.

Elemental mapping of an area with particles showed high relative proportions of O, Na and P and some Ca, Cl and Si. The single, rounded particles mainly contained O, Na and C, while the N signal was barely detectable in the N map (Fig. 5a,b, Supplementary Fig. 15).

STEM-EDS elemental analysis of isolated roundish particles (Fig. 5a-c and Supplementary Figs. 15, 17) suggests, based on the elements’ stoichiometry, primarily sodium carbonates (Na: 20 ± 3 at.%; C: 23 ± 7 at.%; O: 41 ± 9 at.%). The micrometer-sized rounded particles shown in Fig. 5a presented crystalline reflections in the reciprocal space (Fig. 5d) with d-spacings of 3.19 Å, 2.82 Å, 2.00 Å, 1.62 Å, and 1.27 Å, corresponding to the (220), (202), (− 514), (204), (− 441) crystal planes of monoclinic natron (Na_2_CO_3_·10H_2_O^39,40^) with a matching accuracy of 99.28–99.96% (Fig. 5e, Supplementary Fig. 17 and Table 2). The sodium carbonate particles were preferentially round in shape, but other more angular morphologies were found occasionally within the isolated pellet. It was interesting to note that these carbonate microparticles were highly reactive. Under the electron beam, the carbonate particles degraded rapidly. This degradation could occur due to the low melting point of natron and water release from the crystal. Indeed, SAED reflections corresponding to the d-spacings of thermonatrite (Na_2_CO_3_·H_2_O) and nahcolite (NaHCO_3_) were also detected (Fig. 5e)^41,42^. We measured the carbonic acid concentrations of both the expelled and expelled under mechanical stimulus slime using a carbonic anhydrase enzymatic assay that detects dissolved carbonic acid (HCO_3_^−^), finding values of 2.8 ± 0.3 and 1.4 ± 0.2 wt% respectively, which confirms the presence of carbonates and suggests the decrease during the expulsion process could be related to CO_2_ release. The peak at 1400 cm^−1^ in the expelled slime FTIR spectra ( Supplementary Fig. 8) could be associated with the carbonate functional groups^43^. Several authors havereported that the spider silk’s pH and CO_2_ concentration could be part of the processes occurring to drive the protein structural changes^30,44^. We observed that a similar process occurs in the velvet worm slime as it will be described below.

**Figure 6:**
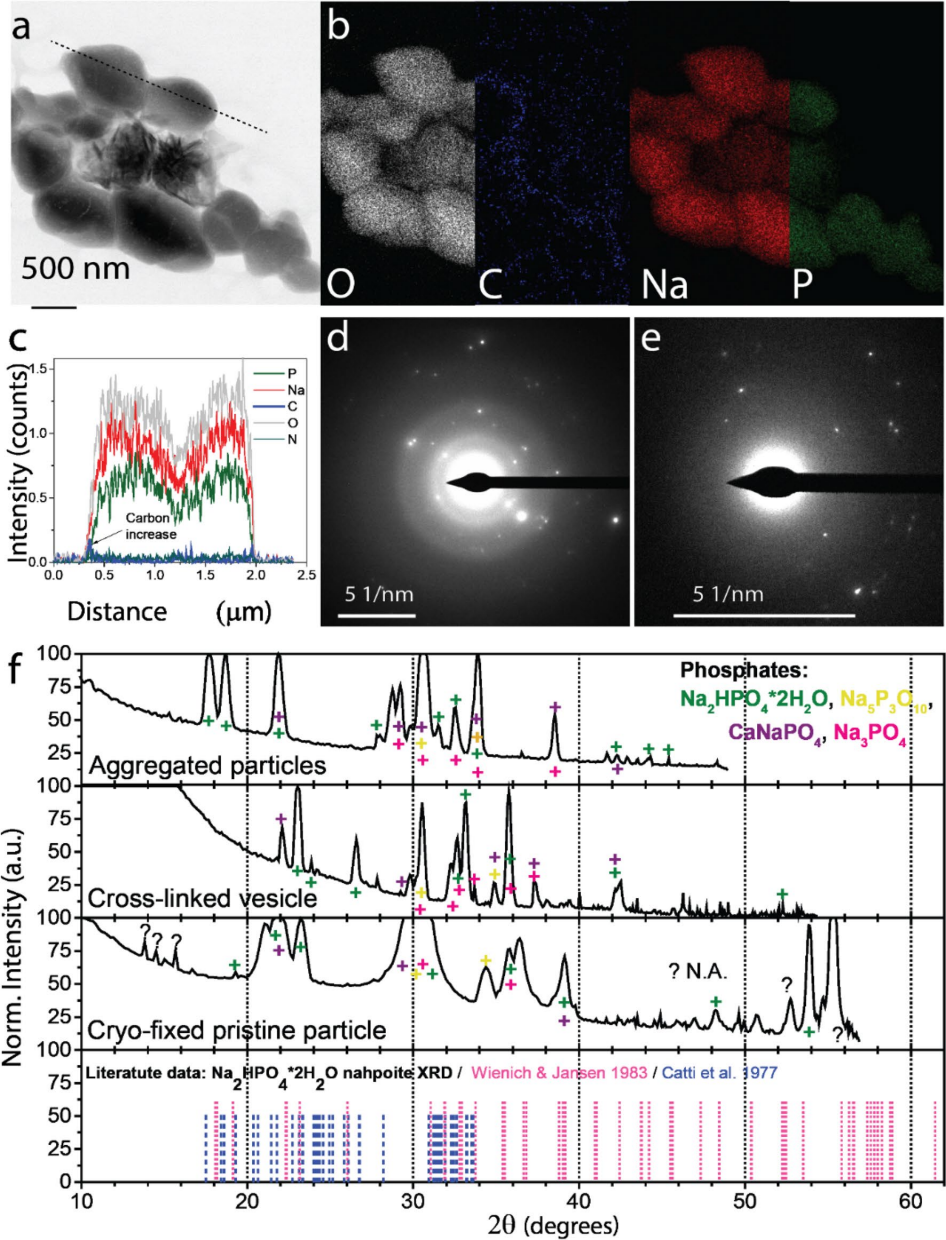
Morphology and chemical composition of phosphate particles in the expelled slime before experiencing an external mechanical stimulus. (**a**) STEM , BF micrograph of isolated polyphosphate globule containing angular phosphate particles. (**b**) Elemental mapping of particles shown a (STEM-EDSX). Elemental mappings of less abundant elements are presented in Supplementary Fig. 18. (**c**) Elemental profile of the dotted line in panel (**a**). Cryo-TEM-selected area electron diffraction (SAED) analysis of (**d**) phosphate particle precipitated from the expelled slime before experiencing an external mechanical stimulus, (**e**) aldehyde-fixed phosphate particles aggregate. (**f**) Crystalline phase analysis based on chemical composition and d-spacings. The color symbols show peaks corresponding to different phosphate species.

The second main group of isolated more angular particles was identified analogously to consist of sodium phosphate Na: 29.1 ± 5.3 at.%; P: 19.2 ± 3 at.%; O: 37 ± 8.3 at.%) (Fig. 6a,b and Supplementary Fig. 18). Silicates and sulfates were also isolated though in smaller quantities (Supplementary Fig. 19). The phosphate particles were mainly grouped in larger globular structures with a carbon-rich layer as shown in Fig. 6a,b. The individual phosphate particles gave rise to crystalline reflections in the reciprocal space with d-spacings of 4.84 Å, 4.57 Å, 3.84 Å and 2.41 Å, corresponding to the crystal planes of hydrated nahpoite (Na_2_HPO_4_·2H_2_O) with a matching accuracy of 99.13–99.89% (Fig. 6d)^45^. Figure 6 f shows that other crystalline phosphate species were also identified, such as CaNaPO_4_ (*e.g.* lit. d-spacing of 2.66, 2.75, 3.80 and 3.84 Å), Na_5_P_3_O_10_ (lit. d-spacings between 2.22–2.67 and 1.45–1.66 Å), Na_3_PO_4_ (*e*.*g*. the d-spacings of 2.62 and 2.64 Å, although the solubility of this salt makes it rather unlikely), and hydroxyapatite Ca_5_(PO_4_)_3_OH (*e*.*g*. lit. d-spacings 4.08 and 3.17 Å) (also refer to Supplementary Table 3 and the lit. XRD spectra)^46^.

The three peaks at 2θ < 20° (5.49, 5.96, 6.26 Å) that did not fit phosphate species could be explained by the fact that the carbon-rich layer on the phosphate particle was partially crystalline. Furthermore, second-order harmonics reflections of the phosphate species could be responsible for the unidentified peaks above 2θ > 50° because the intensity of electron diffractions falls less rapidly with 2θ than the intensity of X-ray diffractions. Moreover, these inorganic species might interact, resulting in other species, such as calcium silicates, apatite, and calcium chloride.

Divalent cations could help to decrease the solubility of phosphate salts in water, as reported by Muller et al. that trigger the coacervate formation of phosphates by adding calcium^47^. Phosphate salts are kosmotropic salts that could control protein suspensions' phase separation and coacervation^48^. AFM studies of the slime reconstituted in water, NaCl, Na_2_CO_3_ and Na_5_P_3_0_10_ at a pH = 7 showed that the protein aggregates’ morphology and size depend on the salts type and the solution pH (Supplementary Fig. 20). The nanoglobules were reversibly formed when NaCl was added to the dialyzed slime solution and tend to aggregate in longer fibers and bigger aggregates when sodium carbonate and tripolyphosphate were added. Bauer et al. showed that the type of salts, ionic concentration and the pH plays a role in the protein aggregation using dynamic light scattering^14^. These results suggest that the carbonic acid and the polyphosphate influence the protein aggregation process and structure besides the shear forces^47–50^. The presence of polyphosphates stored inside vesicles is widely recognized in insects, bacteria, and fungi, among other species^51^. Liu et al.^30^ reviewed that for spider silk, the inorganic compositions account for ∼10 − 20% of the total low molecular weight compounds, containing the dihydrogen phosphate ion (H_2_PO_4_^−^), the potassium ion (K^+^), NO_3_^−^), Na^+^, Cl^−^, and the calcium ion (Ca_2_^+^) and that also the adhesive properties could be affected^30^.

Solubilized carbonates and phosphates can neutralize each other in an acid–base reaction, commonly known as a baking powder reaction. Interestingly, the simultaneously basic and acidic properties of the expelled slime have already been described as early as 1874 when Moseley et al. noted a “slightly bitter and at the same time somewhat astringent” taste of the slime^18^. Carbonates, biological buffers that stabilize alkaline conditions, are known for being extremely reactive with water and acidic solutions^52^. However, further studies are necessary to determine the amount of CO_2_ formation. As the slime has a pH value of 5 that increases up to approximately 7 after dissolving all the particles and mixing the components, some dissolved species that could be formed in the aqueous solution from the Na_2_HPO_4_·2H_2_O solid crystals at this pH are phosphoric acid (H_3_PO_4_ = H^+^  + NaH_2_PO_4_, pK_a_ = 2.14) and sodium dihydrogen phosphate (NaH_2_PO_4_ = H^+^  + Na_2_HPO_4_, pK_a_ = 7.20). Therefore, we propose the following reaction mechanism (not considering the protein contributions) for the reaction of the particles within the liquid media:

$${\text{3Na}}_{{2}} {\text{CO}}_{{3}} \left( {{\text{aq}}.} \right) \, + {\text{3NaH}}_{{2}} {\text{PO}}_{{4}} \left( {{\text{aq}}.} \right) \, + n{\text{X}}^{ + } \left( {{\text{aq}}.} \right) \, + n{\text{Cl}}^{ - } \left( {{\text{aq}}.} \right) \to {\mathbf{3CO}}_{{\mathbf{2}}} \left( {\mathbf{g}} \right) + {\text{ 3Na}}_{{{3} - {\text{n}}}} {\text{X}}_{{\text{n}}} {\text{PO}}_{{4}} \left( {{\text{s}}./{\text{aq}}.} \right) + {\text{ 3H}}_{{2}} {\text{O }}\left( {\text{l}} \right) + n{\text{NaCl }}\left( {{\text{s}}./{\text{aq}}.} \right)$$

$${\text{X}} = {\text{ H}},{\text{ Na}},{\text{ K or }}\raise.5ex\hbox{$\scriptstyle 1$}\kern-.1em/ \kern-.15em\lower.25ex\hbox{$\scriptstyle 2$} {\text{ Ca}}; n = 0 - {2};$$

In this reaction, X has got a nominal charge of + 1 and represents freely dissolved cations that eventually serve as the counterions of the partially insoluble phosphate reaction products. The d-spacings consistent with the reactants and reaction products were found in both carbonate and phosphate particles (Figs. 5, 6).

The release of basic ions accompanies the observed release of CO_2_ gas from the carbonates and the formation of highly soluble phosphate bases (Na_3−n_X_n_PO_4_, Na_2−m_X_m_HPO_4_), which can explain the observed increase in pH in the slime from five to approximately 7. The solid Ca_5_(PO_4_)_3_OH (apatite) is consistent with the electron-dense structures co-located with Ca, P and O seen mainly in the isolated pellet and likely a reaction side product. The above reaction suggests that excess solid NaCl crystals remain as particles in the dried slime. Indeed, we observed numerous NaCl crystals in TEM-EDS as highly electron-dense, cubic, or angular nanoparticles.

The role of silicate ions in the slime remains less clear than that of carbonates and phosphates, even though it can be hypothesized that the former will likely allow for cross-linking of fibrous proteins in the slime.

### Influence of the salt on slime Young’s modulus, tension pull-off adhesion force and rheological properties

**Figure 7:**
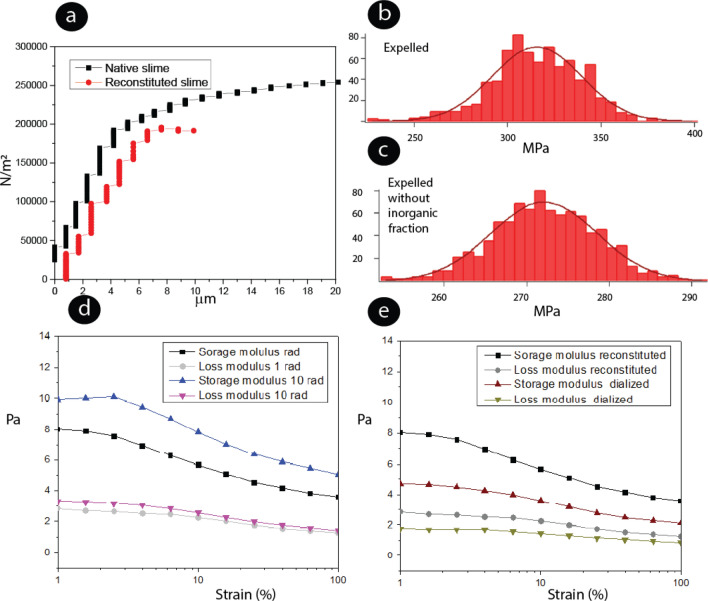
Rheological characterization of expelled slime. (**a**) Effect salt remotion on the slime’s pull-off adhesion force (**b**) and (**c**) Young's modulus calculated by AFM nanoindentation. The mass of the native and non-dialyzed slime was approximately 0,8 mg. Histograms from the expelled slime and expelled without the inorganic fraction. (**d**) Storage and loss modulus of the slime reconstituted in acetic acid 0.01 wt% and dialyzed at different frequencies, concentration 40 mg/mL. The acetic acid at 0.1 wt % was used initially to solubilize the slime and further diluted with water.

The tension adhesion pull-off force of two glass blocks glued with the native, reconstituted, and dialyzed slime was compared. The native slime pull-off force of the sample glued with a slime dried mass of 1.1 mg was 251 kPa, and the material was stretched 20 µm before failure, Fig. 7a. The reconstituted slime pull-off force was 193 kPa, and the dislocation was 7.64 µm before failure, Fig. 7a. The samples glued with the dialyzed slime fell apart immediately, and the adhesion pull-off force could not be measured. The proteins tended to precipitate and separate from the solution after dialysis. The salts and small molecular weight compounds could affect the protein solvation and stabilization in solution affecting the adhesion properties^53^. We observed that the reconstituted slime did not recover the adhesion and fast drying properties as compared to the native slime, suggesting that the initial crystalline molecular arrangement plays a critical role in the adhesion process and drying.

Young’s modulus of the slime without the salts and low molecular compounds and the expelled slime were measured. The tendency from five independent sample measurements is shown in Fig. 7b,c. The expelled slime (average 315 ± 45 MPa) shows a higher Young Modulus than the matrix without the inorganic fraction (average 272 ± 26 MPa), suggesting the inorganic salts' role in the material's cross-linking and hardening^54^.

The results of the dynamic oscillation tests gave important information concerning the effect of the salts and low molecular weight compounds on the slime behavior. Strain sweeps confirmed the gel-like character from the native slime reconstituted solution, as the elastic modulus G′ was higher than the viscous modulus G″ in the linear viscoelastic regime (Fig. 7d). The native slime had a higher G′ value, suggesting a stronger gel with a solid-like character that dominates over the viscous portion. The storage modulus increased when the frequency was increased (Fig. 7d). This is associated with the rate of shear increase, which is proportional to the energy input to the biomolecules. The G′ was lower for the dialyzed slime, suggesting that the salts could influence the intermolecular associative interactions^55–57^. Furthermore, the storage modulus continues to dominate the loss modulus, but the difference between them is smaller, suggesting that the salts and small molecular weight compounds may promote the network gel formation, Fig. 7e^58^.

## Conclusions

Our findings achieved by high-resolution and time-dependent physicochemical characterization of the velvet worms’ slime reveal structural and chemical alterations following the impact of mechanical stimuli. Alterations of the visual appearance (turbid to transparent) and the pH value (acidic to neutral or slightly basic) correlate with a microstructural transformation starting from aggregates and globules containing particles high in C, O, P, Na, Ca and Cl before the expulsion, and characterized by the disappearance of the rounded carbonate and angular phosphate particles in the expelled slime. Several independent lines of evidence suggest that during and after the expelling process and mechanical stimulus, micro- and nanoparticles previously stabilized by a lipid or protein coat dissolve and react with each other in the acidic slime matrix. Evaluation of crystal diffraction signals confirmed the chemical identity of natron and nahpoite with excellent matching accuracy. The increase of both ionic strength and pH is known to favor coextensive fibrous protein connections and assembly. The dispersed salts and low molecular weight compounds also enhance the slime’s gel-like behavior and its glassy appearance and increase Young’s modulus and adhesion force.

Overall, these results point toward a multi-stage mechanism for the gelation and hardening of the slime. The slime appears to be stored in a liquid-crystalline state in the reservoir at an acidic pH. During expulsion, the inorganic carbonate and phosphate particles disappeared and thus must have reacted with the liquid slime and with each other. This increased the expelled slime’s pH level, produced CO_2_, and increased the ionic strength. These changes in the slime could subsequently drive, together with the shear stress, the protein aggregation, phase separation, drying and hardening of the slime and lead to the resulting sturdy composite biopolymer. Further studies are necessary to determine the functions of other observed salts in the velvet worm slime, the underlying mechanism for the particle biomineralization process, and the ways how the worm could store the proteins in a liquid crystalline phase. The roles of the glycoproteins and (lipo) protein nanoparticles as potential natural lubricants within the slime also need further investigation.

The mechanism of the rapid polymer curing presented here through lipid-protein-stabilized carbonate and phosphate microparticles may be used to design new industrial polymerization processes in which aqueous formulations of polymers are, for example, injection-molded, serve as biocompatible chemo-mechano-responsive adhesives, or are used for intravital bioprinting. Another interesting application could be the design of bio-inks that change stiffness as a function of shear forces applied during the printing process.

## Methods

### Worm and slime collection, specimen dissection

Specimens of the species *Epiperipatus biolleyi* were collected in Las Nubes de Coronado, Costa Rica, under the permission R-001-2018-OT-CONAGEBIO provided by the Costa Rican authority CONAGEBIO (Comisión Nacional para la Gestión de la Biodiversidad). Ten female velvet worms were dissected. The observation of a placenta confirmed the sex. Before dissection, the worms were anesthetized by placing them into a carbon dioxide chamber for 10 min and then euthanized in a dissection glass Petri dish containing cotton impregnated with chloroform. The slime reservoir was then extracted and opened, allowing the liquid, unexpelled slime to flow out (Supplementary Fig. 1). slime collected following the expulsion from the oral papilla as described by Mora et al.^11^ and after an external mechanical stimulus is referred to herein as “expelled slime”. Most of the characterization analysis were performed inmediately after collecting the slime to avoid bacterial growth. Even dough, it was previously reported the presence of antimicrobial peptides that protect the slime of bacterial growth^10^.

### Slime water content and slime film stability

The water content of the liquid unexpelled and expelled slime before mechanical stimulus (Fig. 1) was determined by weighing the slime before and after drying using a microbalance (BOECO, Germany). Both liquid and dried unexpelled slime appeared turbid under white light. A pH paper indicator was used to determine the pH of the native expelled slime, and the expelled slime rehydrated to its original volume using ultrapure water (Milli-Q, 18.2 MΩ arium 611 DI, Sartorius Stedim Biotech, Germany).

An enzymatic assay (MaxDiscovery™, Bioo Life Science Products, USA) served to detect carbonic acid (HCO_3_^−^) from dissolved carbonate particles within the slime. The carbonic acid was quantified in 2.8 mg of dried expelled without movement and expelled slime dissolved in 60 µL of CO_2_-free water (supplied in the MaxDiscovery kit). T

We performed stability tests of solidified threads of expelled slime on glass by submerging them in acetic acid (0.1%, pH = 5) and 100 mM PBS (pH = 7.4) for 1 h and then rinsing them with Milli-Q water.

### Electron microscopy

Elemental distribution maps were recorded in scanning transmission mode on a Talos F200X (Super-X EDS, 4-detector configuration, FEI, USA) at an accelerating voltage of 200 kV. Samples were mounted on a double-tilt holder and fixed using a brass ring and clamp. The data were processed using the software Velox 2.9 (FEI, USA). For the maps, a minimum of 17 frames was recorded with a dwell time of 10 µs per pixel unless noted otherwise. To minimize electron-beam-induced carbon deposition and beam-induced degradation of the particles that were highly sensitive to beam damage, the sample shown in Figs. 4, 5 were mounted on a liquid nitrogen-cooled TEM holder (Gatan Inc., US).

The samples for elemental analysis and quantification of particles (Supplementary Table 2) by SEM–EDS and elemental quantification were non-sputtered. The SEM images (18 kV) measurements were obtained using a TESCAN Mira 3 LM field emission instrument with an Octane Pro EDS detector (METEK, USA). The slime was deposited on freshly cleaved highly oriented pyrolytic graphite (HOPG) for these measurements.

For cryo-electron microscopy (cryo-TEM), a drop of fresh liquid unexpelled or expelled slime was added directly onto a lacey carbon film (PlanoEM, Jena, Germany). Excess material was blotted with filter paper (Whatman 40, Little Chalfont, United Kingdom) for 5 s. Then, the sample was immediately plunged into a eutectic mixture of propane and ethane (Carbagas, Switzerland) using a home-built guillotine-type cryoplunger. The vitrified sample was then loaded into the cryo-transfer holder (model Gatan 626 single tilt). Cryo-TEM images were obtained using a Tecnai Spirit TEM operating at 120 kV equipped with a bottom-mount Eagle camera (both FEI Thermo Fischer, Hillsboro, Oregon, US) not exceeding a dose of 100 electrons Å^−2^.

### Isolation and characterization of micro- and nanostructures in the unexpelled and expelled slime

The slime was slowly added to a 1.5 mL Eppendorf tube, and the suspension was centrifugated to 5000 rpm. The pellets were redispersed in 10 µL of Milli Q water, respectively. Five µL of the final dispersions were air-dried at room temperature directly on highly oriented pyrolytic graphite substrates for SEM and on TEM grids for STEM. For protein and lipid staining of the micro- and nanostructures from the pristine expelled or unexpelled slime, paraformaldehyde 2 wt% (Sigma-Aldrich, Germany) was added to the liquid slime (5 µL of paraformaldehyde solution: 100 µL of slime volume) and allowed to react for 15 min. The liquid remained white after the addition of the cross-linking solution. The suspension was centrifuged at 5000 rpm for 10 min, and the pellet was separated from the liquid supernatant. The pellets were redispersed in 10 µL of Milli Q water, respectively. Five µL of the final dispersions were air-dried at room temperature directly on freshly cleaved highly oriented pyrolytic graphite substrates for SEM and on TEM grids for STEM. Dried pellets and supernatant solutions were analyzed using Fourier-transform infrared spectroscopy (FTIR). The spectra were measured on a Bruker Vertex 70 with an IR Scope II extension.

### Protein staining

Fluorescence staining of micro- and nanostructures was performed within the expelled slime and isolated particles. The micro- and nanostructures in the expelled slime and isolated particles were stained using the following dyes: rhodamine B isothiocyanate (RITC) at 4 µM in 0.1 M PBS (Sigma-Aldrich, Germany) for proteins; the lipids with CellMask™ Deep Red Plasma membrane stain at 10 µM in PBS (Thermo Fisher Scientific, Germany). Brightfield microscopy and fluorescence images were taken using a laser-scanning microscope (LSM 710, Zeiss, Germany).

### Attenuated total reflectance- Fourier transform infrared (ATR-FTIR)

FTIR spectra were recorded on a Bruker ALPHA II series spectrometer equipped with an ALPHA’s Platinum ATR single reflection diamond ATR module. Spectra were averaged over 32 scans using a resolution of 2 cm^−1^ over the IR range of 4000–400 cm^−1^. The data were analyzed using the software Bruker OPUS (version 8.1).

### Amplitude-modulated atomic force microscopy (AFM)

Sample topography was analyzed in the air using an AFM microscope (Asylum Research, Santa Barbara, CA) operated in tapping mode. Silicon probes (model Tap150Al-G), backside with resonance frequencies of 150 kHz and force constant of 5 N/m were used. AFM was operated in contact mode silicon tips with a nominal tip radius of 10 nm and cantilever spring constant k = 13 N/m (VEECO) for obtaining the force curves. The spring constant was calibrated according to the supplier protocol and using the Asylum Research Software version 15.09.112 and the GetReal function. The data was modeled using the Hertz model, assuming a 10 nm silicon (100) tip and a material passion ratio of 0.33 common value for polyacrylamide gels. The area analyzed was 1 µm. The Hertz Model was used to model the data.

### Tension pull-off adhesion force of the native and dialyzed slime

**Figure 8:**
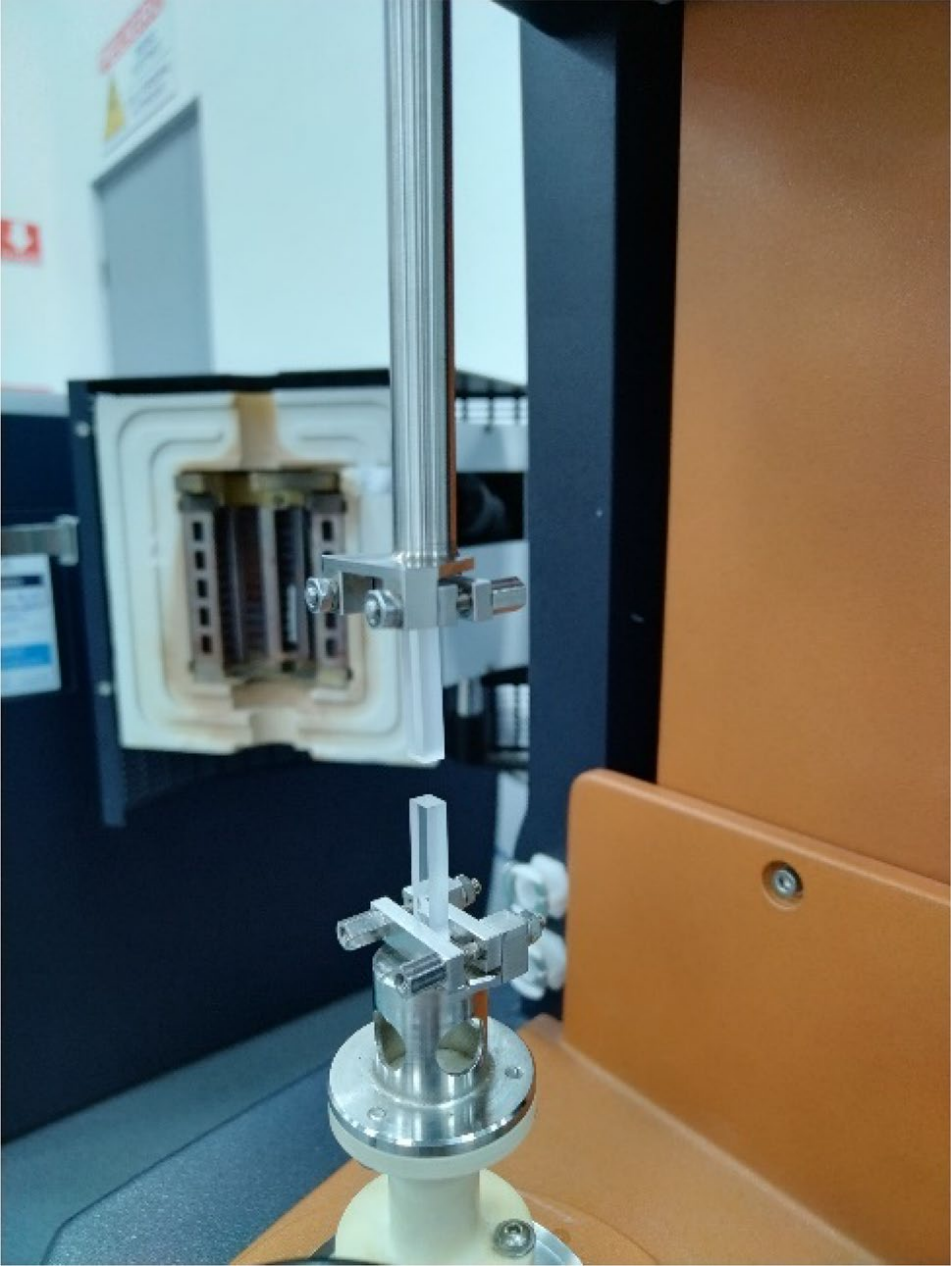
Photograph (taken by the authors) of the pull-off assembly glass blocks used clamped to the rectangular geometry from the DMTA (Discovery HR-3/Hybrid Rheometer, TA Company).

The effect of the salt removal on the adhesion pull-off force was determined by gluing the smaller side area of two rectangular glass blocks with 5 cm × 3.5 cm, Fig. 8. Initially, the two glass blocks were pre-weighted and a drop of the native slime was added on top of one of the lower block surface that was previously clamped. The upper glass block was slowly lowered until it made contact. The system was kept for one hour, and then the upper block was pulled away at a constant speed. The dried glass blocks were weighed again to determine the slime mass to prepare the glass block glued with a similar amount of the reconstituted slime and desalted sample. The weighted mass for the three repetitions was 0.7, 1, and 1.1 mg. The load − displacement behavior was recorded every 0.001 s. The force just before the slime released contact between the two glass blocks was recorded. A universal test machine (Discovery HR-3/Hybrid Rheometer, TA Company) with rectangular torsional geometry was used. The pull-off value reported is the pull of force divided by area.

### Rheological properties of the slime with remaining salts and dialyzed

Rheological measurements were carried out in a universal test machine (Discovery HR-3/Hybrid Rheometer, TA Company) and using parallel-plate Geometry in a steady and oscillatory shear with plate–plate geometry (25 mm diameter, gap of 0.5 mm) at 20 °C. 500 µL of the samples were carefully pipetted on the lower plate, and the upper cone was then lowered to a 0.052 mm gap distance. Prior to the test, the samples were left to equilibrate for five minutes. Storage (G′) and loss (G″) moduli were recorded as a function of the strain, in a range between 0.1 and 100%, at a constant angular frequency of 1 and 10 rad/s and at a fixed temperature of 20 °C. A solution of 40 mg/mL of the reconstituted slime and the same samples after dialysis with a dialysis tubing cellulose membrane, molecular weight cut-off 12,000–14,000 (Sigma, Germany), was used for the experiments.

## Supplementary Information

Supplementary Information.

Supplementary Video 1.

Supplementary Video 2.

## Data Availability

Correspondence and requests for materials should be addressed to YCU.

## References

[1] Yang SY (2013). A bio-inspired swellable microneedle adhesive for mechanical interlocking with tissue. Nat. Commun..

[2] Lutz TM, Kimna C, Casini A, Lieleg O (2022). Bio-based and bio-inspired adhesives from animals and plants for biomedical applications. Mater. Today Bio.

[3] Aldred N (2020). Chitin is a functional component of the larval adhesive of barnacles. Commun. Biol..

[4] Pablo Barquero-González J, Vega-Hidalgo Á, Monge-Nájera J (2019). Feeding behavior of Costa Rican velvet worms: Food hiding, parental feeding investment and ontogenetic diet shift (Onychophora: Peripatidae). Cuad. Investig. UNED.

[5] Monge-Nájera J (2021). Onychophorology, the study of velvet worms, historical trends, landmarks and researchers from 1826 to 2020 (a literature review). Uniciencia.

[6] Pablo Barquero-González J, Alonso Cabrera Alvarado A, Valle-Cubero S, Monge-Nájera J, Morera-Brenes B (2016). The geographic distribution of Costa Rican velvet worms (Onychophora: Peripatidae). Rev. Biol. Trop..

[7] Concha A (2015). Oscillation of the velvet worm slime jet by passive hydrodynamic instability. Nat. Commun..

[8] Corrales-Urena YR (2017). Extracellular micro and nanostructures forming the velvet worm solidified adhesive secretion. Mater. Res. Express.

[9] Baer A (2017). Mechanoresponsive lipid-protein nanoglobules facilitate reversible fibre formation in velvet worm slime. Nat. Commun..

[10] Haritos VS (2010). Harnessing disorder: Onychophorans use highly unstructured proteins, not silks, for prey capture. Proc. R. Soc. B.

[11] Benkendorff K, Beardmore K, Gooley AA, Packer NH, Tait NN (1999). Characterisation of the slime gland secretion from the peripatus, *Euperipatoides kanangrensis* (Onychophora: Peripatopsidae). Comp. Biochem. Physiol. Part B.

[12] Mora M, Herrera A, León P (1996). Análisis electroforético de las secreciones adhesivas de onicóforos del género Epiperipatus. Rev Biol Trop.

[13] Jerez-Jaimes JH, Catalina Bernal-Pérez M (2009). Taxonomía de onicóforos de Santander, Colombia y termogravimetría, calorimetría de barrido diferencial y espectroscopía infrarroja de la secreción adhesiva (Onychophora: Peripatidae). Rev. Biol. Trop..

[14] Baer A, Hänsch S, Mayer G, Harrington MJ, Schmidt S (2018). Reversible supramolecular assembly of velvet worm adhesive fibers via electrostatic interactions of charged phosphoproteins. Biomacromolecules.

[15] Baer A (2019). Shear-induced β-crystallite unfolding in condensed phase nanodroplets promotes fiber formation in a biological adhesive. ACS Nano.

[16] Lu Y (2022). Complete sequences of the velvet worm slime proteins reveal that slime formation is enabled by disulfide bonds and intrinsically disordered regions. Adv. Sci..

[17] Harrington MJ, Fratzl P (2021). Natural load-bearing protein materials. Prog. Mater. Sci..

[18] Robson EA, Lockwood APM, Ralph R (1966). Composition of the blood in onychophora. Nature.

[19] Campiglia SS (1976). The blood of *Peripatus acacioi* marcus & marcus (Onychophora). III. The ionic composition of the hemolymph. Comp. Biochem. Physiol. A Comp. Physiol..

[20] Baer A, Mayer G (2012). Comparative anatomy of slime glands in onychophora (velvet worms). J. Morphol..

[21] Lavallard R, Campiglia S (1971). Données cytochimiques et ultrastructurales sur les tubes sécréteurs des glandes de la glu chez *Peripatus acacioi* Marcus et Marcus (Onychophore). Z. Zelforsch..

[22] Verhoef JJF (2017). Polyphosphate nanoparticles on the platelet surface trigger contact system activation. Blood.

[23] Sutherland TD, Young JH, Weisman S, Hayashi CY, Merritt DJ (2010). Insect silk: One name, many materials. Annu. Rev. Entomol..

[24] Walker AA, Holland C, Sutherland TD (2015). More than one way to spin a crystallite: Multiple trajectories through liquid crystallinity to solid silk. Proc. R. Soc. B.

[25] Sung B, Kim MH (2018). Liquid-crystalline nanoarchitectures for tissue engineering. Beilstein J. Nanotechnol..

[26] Huang Z (2017). A general patterning approach by manipulating the evolution of two-dimensional liquid foams. Nat. Commun..

[27] Mohammadi P (2018). Phase transitions as intermediate steps in the formation of molecularly engineered protein fibers. Commun. Biol..

[28] Baird G (2020). FTIR spectroscopy detects intermolecular β-sheet formation above the high temperature Tm for two monoclonal antibodies. Protein J..

[29] He Y (2018). Lipids as integral components in mussel adhesion. Soft Matter.

[30] Liu X (2021). Recent progress of spider-silk-inspired adhesive materials. ACS Mater. Lett..

[31] Amarpuri G, Dhopatkar N, Blackledge TA, Dhinojwala A (2022). Molecular Changes in spider viscid glue as a function of relative humidity revealed using infrared spectroscopy. ACS Biomater. Sci. Eng..

[32] van Bockstaele F, Romanus M, Penagos IA, Dewettinck K, Toro-Vazquez JF (2022). Chapter 9 Functionality of natural waxes in hybrid fat crystal networks. Development of Trans-Free Lipid Systems and their use in Food Products.

[33] Yang H, Yang S, Kong J, Dong A, Yu S (2015). Obtaining information about protein secondary structures in aqueous solution using Fourier transform IR spectroscopy. Nat. Protoc..

[34] Vahur S, Teearu A, Peets P, Joosu L, Leito I (2016). ATR-FT-IR spectral collection of conservation materials in the extended region of 4000–80 cm–1. Anal. Bioanal. Chem..

[35] Krishnamurthy R, Rajasekaran R, Samuel BS (2013). Growth and characterization of KDP crystals doped with l-aspartic acid. Spectrochim. Acta A Mol. Biomol. Spectrosc..

[36] Karthein R (1988). A Fourier-transform infrared spectroscopic study of the phosphoserine residues in hen egg phosvitin and ovalbumin. Biochem. Biophys. Res. Commun..

[37] Corrales-Ureña YR (2018). Biogenic silica-based microparticles obtained as a sub-product of the nanocellulose extraction process from pineapple peels. Sci. Rep..

[38] Jehle F, Macías-Sánchez E, Fratzl P, Bertinetti L, Harrington MJ (2020). Hierarchically-structured metalloprotein composite coatings biofabricated from co-existing condensed liquid phases. Nat. Commun..

[39] Taga T (1969). The crystal lattice of sodium bicarbonate, NaHCO3. Acta Crystallogr. B.

[40] Zachariasen WH (1933). The crystal lattice of sodium bicarbonate, NaHCO_3_. J. Chem. Phys..

[41] Barthelmy, D. Nahcolite Mineral Data. General Nahcolite Information (2019).

[42] Americal Mineralogist Data Dase (2019).

[43] Kiefer J, Strk A, Kiefer AL, Glade H (2018). Infrared spectroscopic analysis of the inorganic deposits from water in domestic and technical heat exchangers. Energies.

[44] Shang L (2019). Spinning and applications of bioinspired fiber systems. ACS Nano.

[45] Davies DR, Corbridge DEC (1958). The crystal structure of sodium triphosphate, Na5P3O10, phase II. Acta Crystallogr..

[46] Suchanek W, Yashima M, Kakihana M, Yoshimura M (1998). Rhenanite (–NaCaPO_4_) as weak interphase for hydroxyapatite ceramics. J. Eur. Ceram. Soc..

[47] Müller WEG (2020). Nanoparticle-directed and ionically forced polyphosphate coacervation: A versatile and reversible core–shell system for drug delivery. Sci. Rep..

[48] Mohammadi P (2020). Controllable coacervation of recombinantly produced spider silk protein using kosmotropic salts. J. Colloid Interface Sci..

[49] Bashir Z (2022). Engineering bio-adhesives based on protein-polysaccharide phase separation. Int. J. Mol. Sci..

[50] Blocher McTigue WC, Perry SL (2020). Protein encapsulation using complex coacervates: What nature has to teach us. Small.

[51] Ramos I (2011). Acidocalcisomes as calcium- and polyphosphate-storage compartments during embryogenesis of the insect Rhodnius prolixus stahl. PLoS One.

[52] Joshi S, Kalyanasundaram S, Balasubramanian V (2013). Quantitative analysis of sodium carbonate and sodium bicarbonate in solid mixtures using Fourier transform infrared spectroscopy (FT-IR). Appl. Spectrosc..

[53] Sahni V (2014). Direct solvation of glycoproteins by salts in spider silk glues enhances adhesion and helps to explain the evolution of modern spider orb webs. Biomacromolecules.

[54] Braun M, Menges M, Opoku F, Smith AM (2013). The relative contribution of calcium, zinc and oxidation-based cross-links to the stiffness of Arion subfuscus glue. J. Exp. Biol..

[55] Bergonzi C (2019). Study of 3D-printed chitosan scaffold features after different post-printing gelation processes. Sci. Rep..

[56] Holten-Andersen N (2011). pH-induced metal-ligand cross-links inspired by mussel yield self-healing polymer networks with near-covalent elastic moduli. PNAS.

[57] Flammang P, Lambert A, Bailly P, Hennebert E (2009). Polyphosphoprotein-containing marine adhesives. J. Adhes..

[58] Curnutt A, Smith K, Darrow E, Walters KB (2020). Chemical and microstructural characterization of pH and [Ca^2+^] dependent sol-gel transitions in mucin biopolymer. Sci. Rep..

