# Supplementary material for: Encapsulated salts in velvet worm slime drive its hardening
Source: Sci Rep. 2022 Nov 10;12:19261. doi: 10.1038/s41598-022-23523-z (PMC9649676; doi:10.1038/s41598-022-23523-z)
Supplement: Supplementary file 1 — Supplementary Information. [file 41598_2022_23523_MOESM1_ESM.docx]

**Supplementary information**

**Encapsulated salts in velvet worm slime drive its hardening**

**Yendry Regina Corrales-Ureña^*1,2^, Fabienne Schwab^1^, Efrain Ochoa-Martinez^1^, Miguel Benavides-Acevedo^3^, José Vega-Baudrit^3,4^, Reinaldo Pereira^3^, Klaus Rischka^5^, Paul-Ludwig Michael Noeske^5^, Alexander Gogos^6^, Dimitri Vanhecke^1^,** **Barbara Rothen-Rutishauser^1^, Alke Petri-Fink^†1,7^**

^1^ University of Fribourg, Adolphe Merkle Institute, Chemin des Verdiers 4, 1700 Fribourg, Switzerland

^2^Faculty of Production Engineering, University of Bremen, Am Fallturm 1, D-28359, Bremen, Germany

^3^ National Laboratory of Nanotechnology LANOTEC - National Center of High Technology CeNAT, 1.3 km north of the United States Embassy, San José, Costa Rica

^4^ School of Chemistry, National University, Heredia, Costa Rica

^5^ Adhesive Bonding Technology and Surfaces, Fraunhofer Institute for Manufacturing Technology and Advanced Materials IFAM, Wiener Straße 12, 28359 Bremen, Germany

^6^ EMPA, Swiss Federal Laboratories for Materials Science and Technology, Lerchenfeldstrasse 5, CH-9014 St. Gallen, Switzerland.

^7^ Department of Chemistry, University of Fribourg, Chemin du Musée 9, CH-1700 Fribourg

Corresponding author: [*yencor@uni-bremen.de](mailto:*yencor@uni-bremen.de), [yendry386@hotmail.com](mailto:yendry386@hotmail.com)

**Methods.**

**Inductively Coupled Plasma Optical Emission spectroscopy (ICP-OES) analysis**

In the first step, 100 uL concentrated HNO_3_ was added to the sample and left to react for 1.5h at RT (samples did not dissolve completely). Then, the sample was transferred to a digestion tube. Step 1 was repeated 2 times but without the 1.5h reaction time. Subsequently, the Eppendorf tube was rinsed with 100 µL H_2_O.After the sample was completely transferred to the digestion tube, digestion was carried out in a Turbowave pressurized microwave system (MWS GmbH). After the digestion, 200 µL H_2_O_2_ were added to the sample and left to react until a colorless solution was obtained (in the order of a few minutes) and no gas liberation was observed anymore. Subsequently, the sample was transferred to a pre-weighted PP tube and filled to 10ml and weighed again. Determination of the elemental contents was carried out using a ICP-OES (Agilent 5110, in case of Ca, P, K, and Zn) and a ICP MS (Agilent 7900, in case of Na, Cu, Fe, Si, Mg and Al). A certified multi-element standard solution containing the elements of interest (IV71A, Inorganic Ventures) served as a quality control sample. Recoveries ranged between 92 (Ca) and 106% (Al) clean solutions and not precipitates.

**Slime paraffin embedding**

Samples were fixed adding Karnovsky solution (5% glutaraldehyde, 4% paraformaldehyde, 0.1 M phosphate buffer) for 1 h to the slime. They were let it dry and then, dehydrated through an increasing gradient of 30% v/v, 50% v/v, 70% v/v, 90% v/v, 95% v/v and two changes of 99.9% v/v of ethyl alcohol for 30 min each. The slime is insoluble in ethanol. A solid pelled was obatined After that, they were clarified with ethyl alcohol and xylene in 1:1 ratio and two changes of xylene for 30 min each. Finally, samples were embedded with Paraplast Plus® (Leica®, Wetzlar, Germany) in 1:1 ratio with xylene and two changes of Paraplast Plus® for 40 min each incubated in a stove heated between 60 ºC to 65 ºC. 4 µm sections were obtained with a Minot type rotary microtome (Reichert 820H HistoStat model), placed in a Flotation Bath filled with distilled water heated between 40 ºC to 45 ºC, collected with microscopy slides and incubated in a stove heated between 60 ºC to 65 ºC for 1 h. Slides were stained with Hematoxylin-Eosin to observe general morphology, Masson Trichrome to stain collagen, nuclei, muscle and cytoplasm and Periodic Acid Shiff counter stained with Hematoxylin to stain neutral glycosaminoglycans and nuclei (García, 1993; Prophet et al., 1995). Slides were dehydrated, clarified, and mounted permanently with CV Mount medium (Leica®, Wetzlar, Germany). They were analyzed with a Zeiss AXIO microscope (Jena, Germany) and microphotographs were taken with a DP74 Olympus camera (Tokyo, Japan).

**Reservoir Paraffin embedding and collagen staining**

Each worm specimen has two separate reservoirs on each body side, presenting similar structural features as also reported previously for other species.


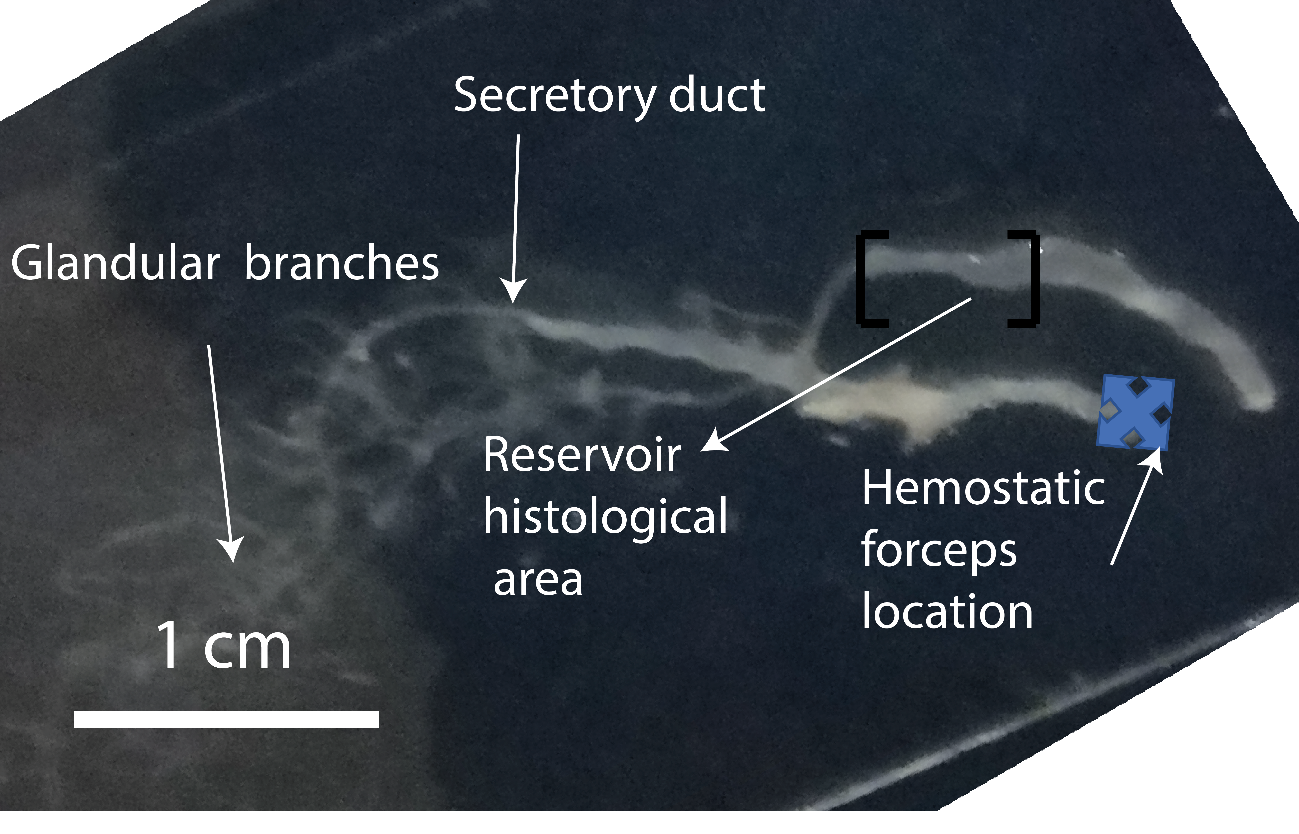


Photograph of the two reservoirs belonged to one specimen. (taken by the auhors)

The reservoir ending was sealed with mosquito hemostatic forceps. After that, samples were fixed in Karnovsky solution (5% glutaraldehyde, 4% paraformaldehyde, 0.1 M phosphate buffer) for 1 h. Then, they were dehydrated through an increasing gradient of 30% v/v, 50% v/v, 70% v/v, 90% v/v, 95% v/v and two changes of 99.9% v/v of ethyl alcohol for 30 min each. Samples were clarified with 99.9% v/v ethyl alcohol and xylene in 1:1 ratio and two changes of xylene for 30 min each. Finally, samples were embedded with Paraplast Plus® (Leica®, Wetzlar, Germany) in 1:1 ratio with xylene and two changes of Paraplast Plus® for 40 min each incubated in a stove heated between 60 ºC to 65 ºC. 4 µm sections were obtained with a Minot type rotary microtome (Reichert 820H HistoStat model), placed in a Flotation bath filled with distilled water heated between 40 ºC to 45 ºC, collected with microscopy slides and incubated in a stove heated between 60 ºC to 65 ºC for 1 h. Slides were stained with H&E to observe general morphology, Masson’s Trichrome to stain collagen, nuclei, muscle, cytoplasm and oxidated structures, periodic acidSchiff counter-stained with Hematoxylin to stain neutral glycosaminoglycans, nuclei, polysaccharides, glycoproteins, and mucins (García, 1993; Prophet et al., 1995). Slides were dehydrated, clarified, and mounted permanently with CV Mount medium (Leica®, Wetzlar, Germany). These were analyzed with a Zeiss AXIO microscope (Jena, Germany) and photomicrographs were taken with a DP74 Olympus camera (Tokyo, Japan).

**Fluorescence staining of the slime and micro- and nanostructures**

Collagen with 1 µM collagen I alpha 1 antibody (Novus, NB600-408) and secondary antibody Rabbit anti-Goat IgG Alexa Fluor™ 594 (Life Technologies, Switzerland) was used. The slime was partially soluble in PBS for at least 1 h, allowing to do the staining and rinsing of the samples without dissolving the slime completely. Brightfield microscopy and fluorescence images were taken using a laser-scanning microscope (LSM 710, Zeiss, Germany). Pierce™ Glycoprotein Staining Kit (Thermofisher, Germany) was used to stain the rinsed pellet following the supplier protocol to determine the presence of glycoproteins.

**Differential Scanning Calorimetry (DSC)**

The glass transition temperature of films was determined using a Q200 differential scanning calorimeter (TA Instruments, USA). The scan was run at 10 °C/min under a nitrogen flow rate of 10 ml/min from 0 to 150 °C . Samples were dried under environmental conditions for 4 days before analysis.

**Thermogravimetric analysis (TGA)**

TGA was performed using a Q500 (TA Instruments, USA). The samples (approx. 5 ± 0.1 mg) were collected in a standard platinum pan. The samples were dried at environmental temperature to avoid degradation before being analyzed. The scan was run at 10 °C/min under a nitrogen flow. Mass change was measured from 50 °C to 800 °C.

**Results and discussions**


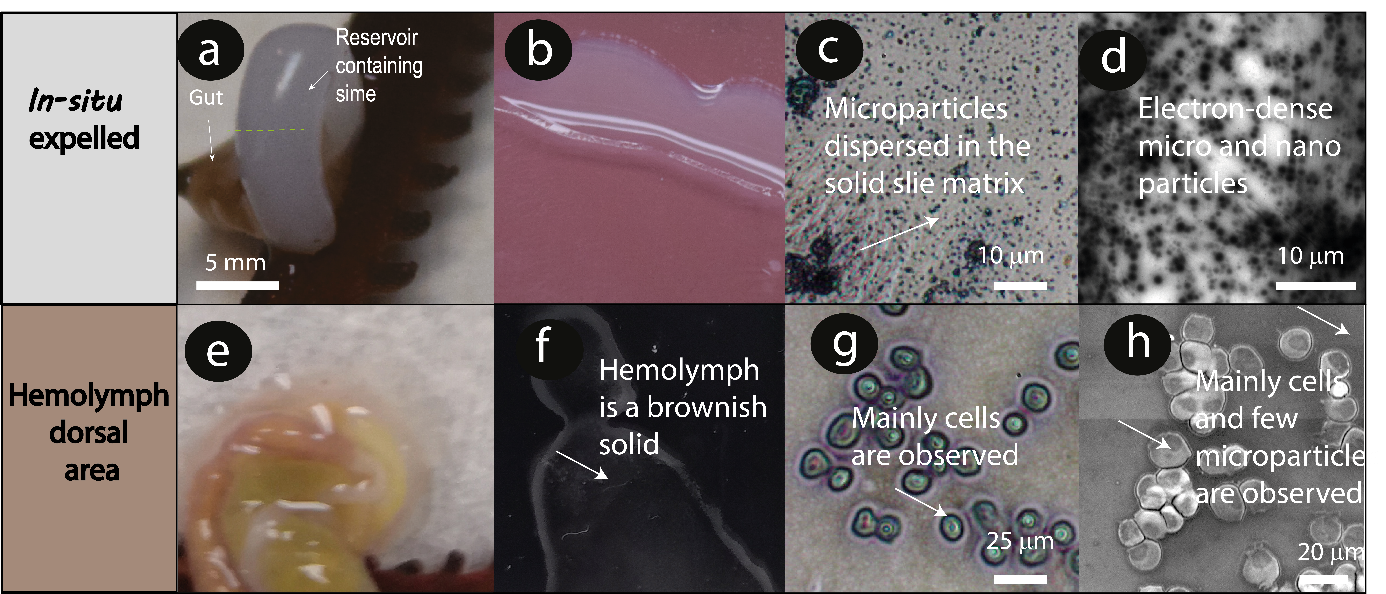


**Figure 1.** Morphology of the slime reservoir. **a** Photograph of the body *from Epiperipatus biolleyi* specimen, note the histological reservoir area under study. Note that the reservoir is semi-full and can be partially separated from the gut without cross-contamination. The hemolymph was removed using a syringe from the dorsal area before separating the reservoir. **b** Photograph of the liquid slime extracted directly from the reservoir. **c** Bright-field micrograph of microparticles dispersed in the unexpelled solid slime. Note that only few cells are present. **d** Electron dense particles dispersed on the solid unexpelled slime that did not experience an external mechanical stimulus. **e** Photograph of the hemolymph surrounding organs. Note the brownish color. **f** Photograph of the dried hemolymph. **g** Bright-field micrograph of the dried hemolymph containing cells. Note that similar particles were not observed surrounding the cells. h SEM image of the dried hemolymph showing mainly cells.


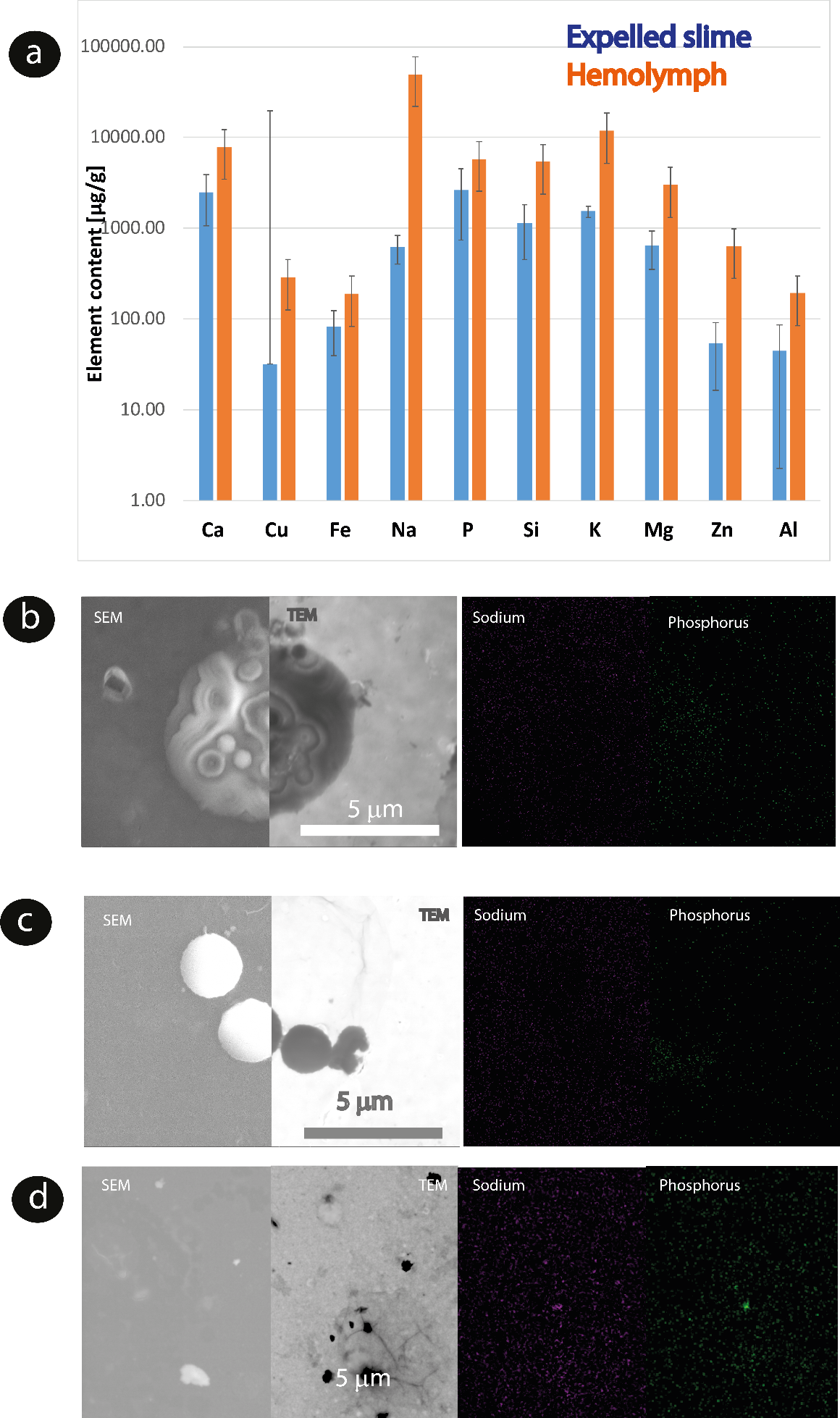


**Figure 2. a** ICP-OES elemental concentration of the slime and hemolymph. **c-d** . STEM-EDS analysis of microstructures contained in the dried hemolymph. Note that the microparticles and cells contains low amount of phosphorous and sodium. Mainly electron-dense calcium and oxygen containing particles were found.

**Table 1.** SEM-EDS results of the hemolymph, expelled slime and pellet.


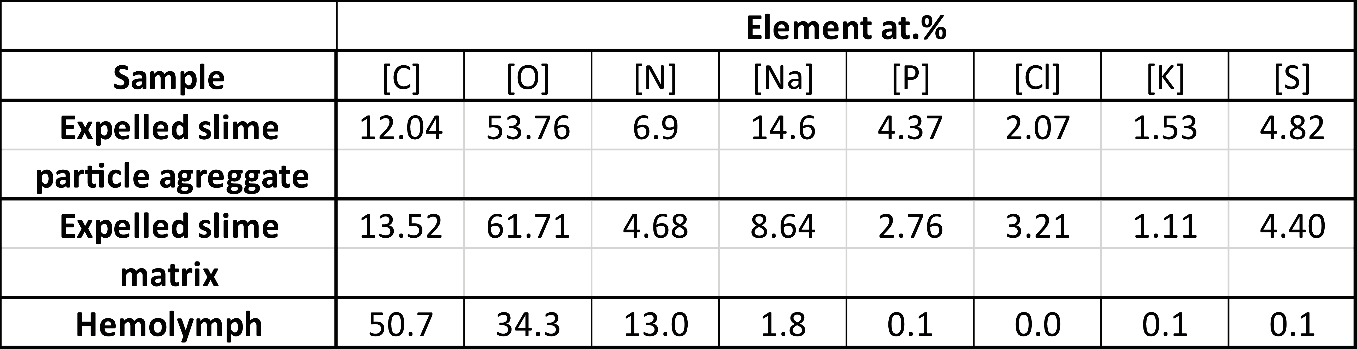


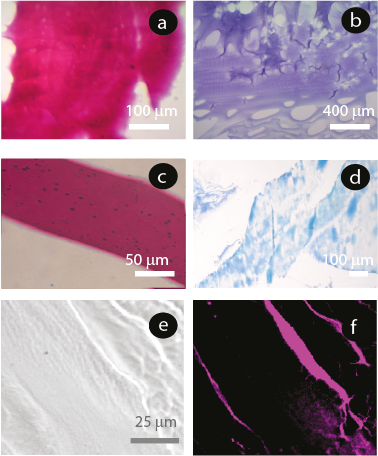


**Figure 3.** **Paraffin s embedded expelled slime sections**. **a** Eosin/hematoxylin staining. **b** PAS. **c** Masson´s staining. Note the blue fibers (dot-like) distributed in the slime matrix **d**. Toluidine staining. Note that the type of glycans that are stored inside the lumen space is not clarified using these staining. Negatively, positively, or neutrally charged glycans could be part of the compositions because of the unspecific staining of the slime matrix. **e** Bright field microscopy image of the expelled slime **f.** Collagen fluorescent immunostaining of area shown in e.


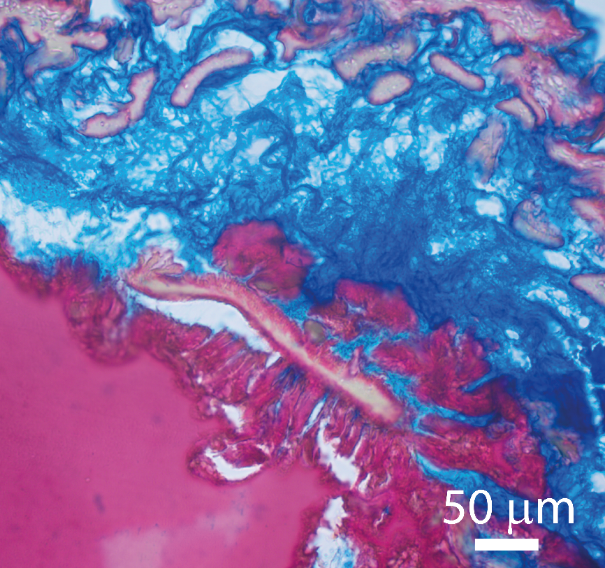


**Figure 4**. **Histological image.** **Cross-section microphotograph of the paraffin-embedded reservoir**. Eosin/hematoxylin staining and Masson stained section, notice how the inner lumen is stained turquoise, while the area between the lumen and the muscle cells is stained blue (the blue color indicates collagen-rich areas).

**
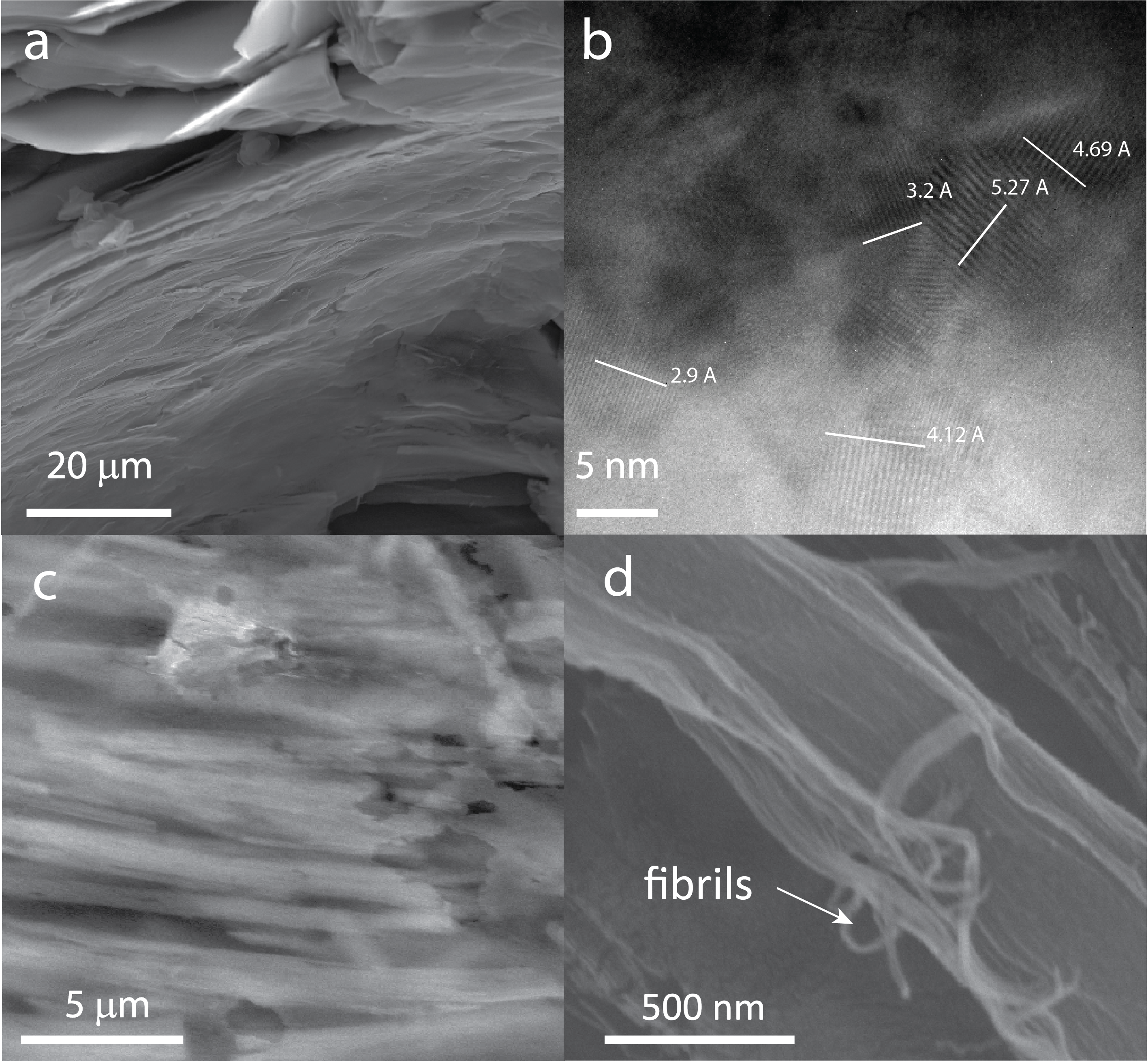
**

**Figure 5. Films of the dried unexpelled slime on HOPG. a** SEM image **b** TEM image of protein matrix**.** The images show a material organized in a lamellar conformation, d-spacing between the protein molecules was calculated for crystalline-like regions.. **c** and **d** Preformed protein fibers. Fibers were composed of nanometer diameter fibrils (SEM images).


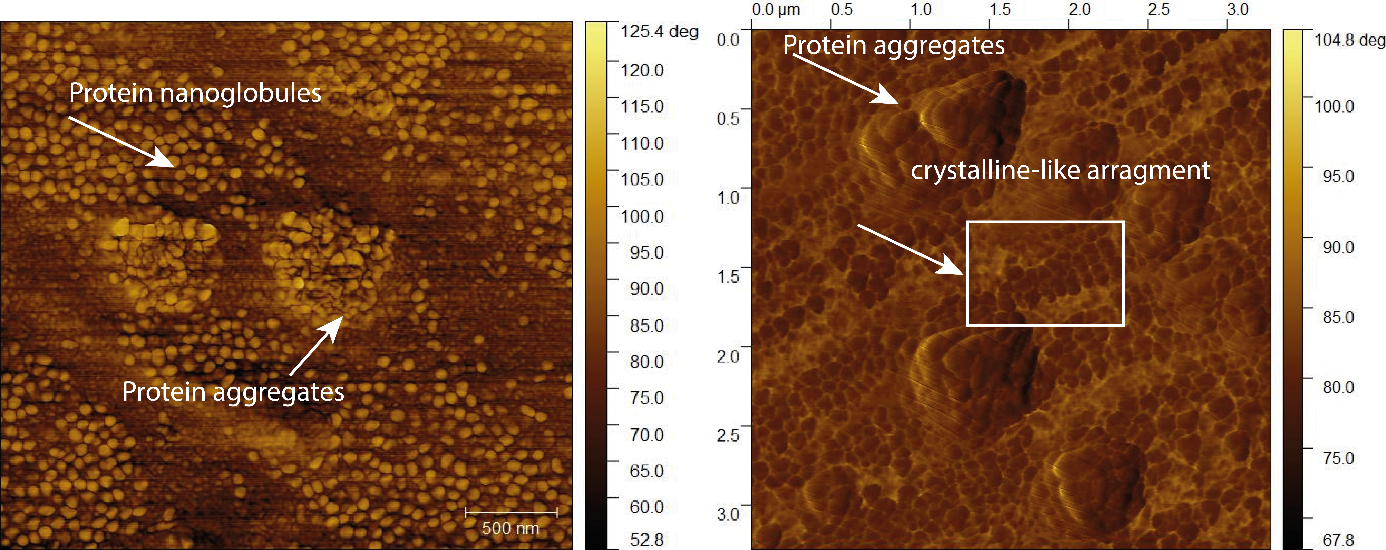


**Figure 6.** a AFM phase images of the dried expelled slime on HOPG. Note that the protein nanoglobules and their aggregates are observed


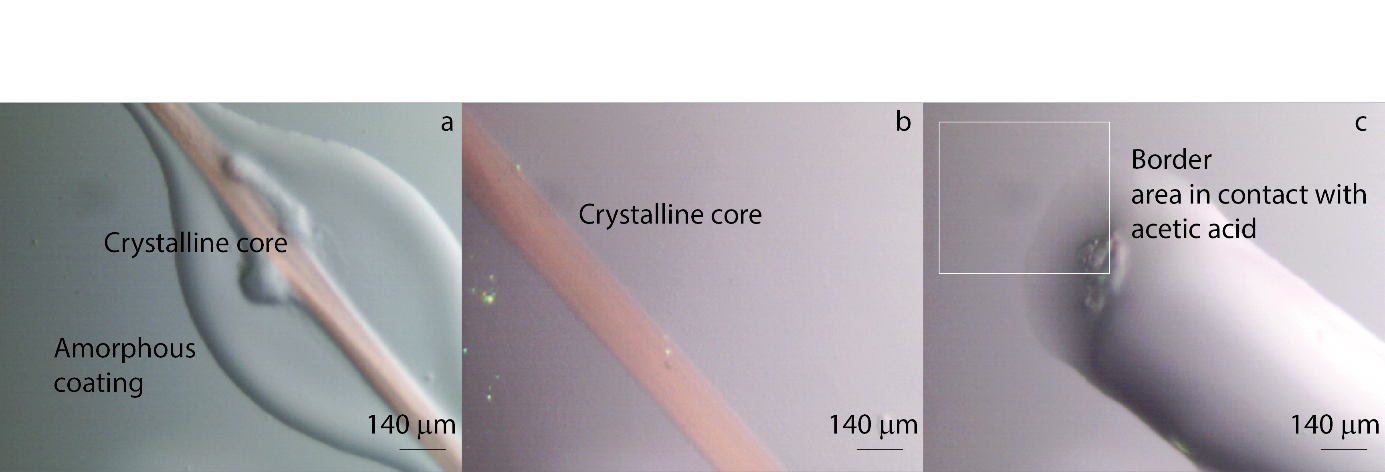


**Figure 7. Polarized microscopy images** a Slime expelled by the worm on glass**, b** a after contact with PBS 100 m M for one hour. Note that the staining of the slime is possible due to its partial stability at pH 7.4. Mainly, the amorphous coating was removed **c** a after contact with acetic acid solution 0.1 wt.% 15 min. Note that the slime is removed from the glass.


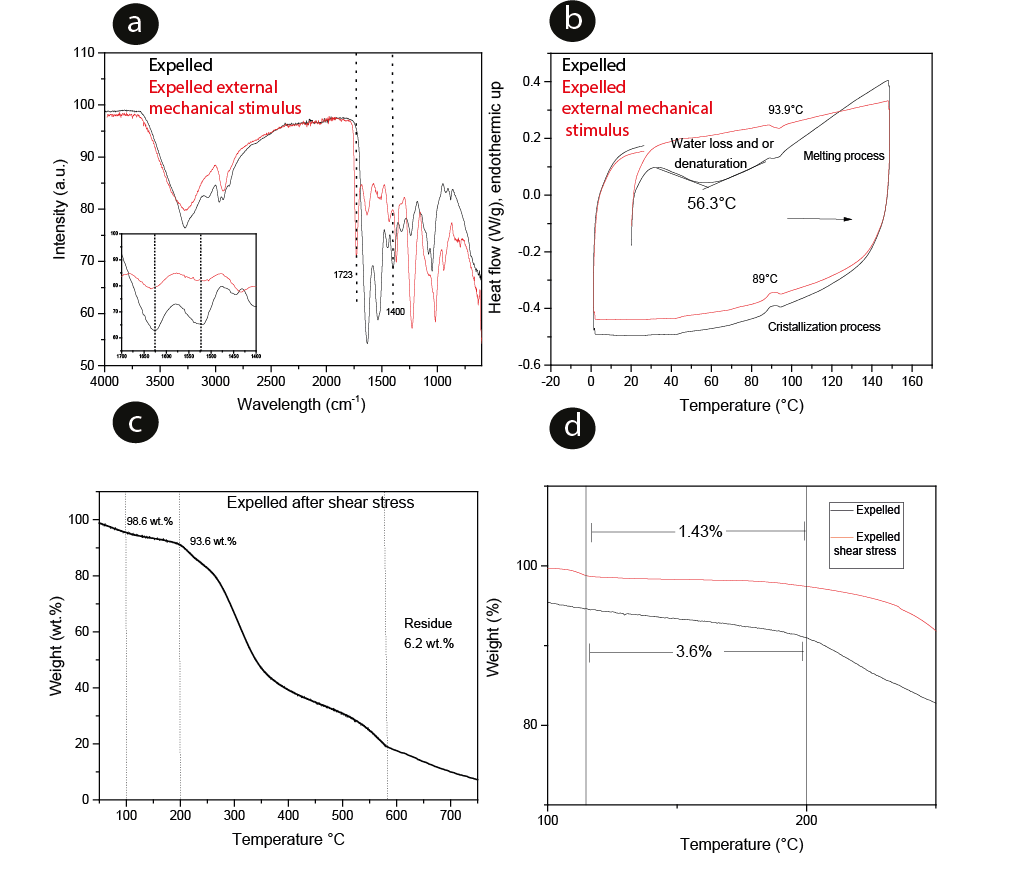


**Figure 8. a** ATR-FTIR spectra of the expelled slime before and after an external mechanical stimulus. **b** DSC measurements of the expelled slime before and after an external mechanical stimulus. **c** TGA thermogram of the slime expelled after an external mechanical stimulation and drying at environmental temperatures. The sample was dried until achieving a stable weight. Note that the slime starts to degrade approximately at 200 °C and there is a final residue of 6,2 wt. % that is correlated to the non-decomposed salts. **d** Comparison of the thermograms of the expelled slime before and after an external mechanical stimulus in the range between 100 °C and 300 °C. Note that the expelled slime accumulated higher amount of water. Also, higher weight loss between 120 °C and 200 °C could be correlated to the carbonate degradation.


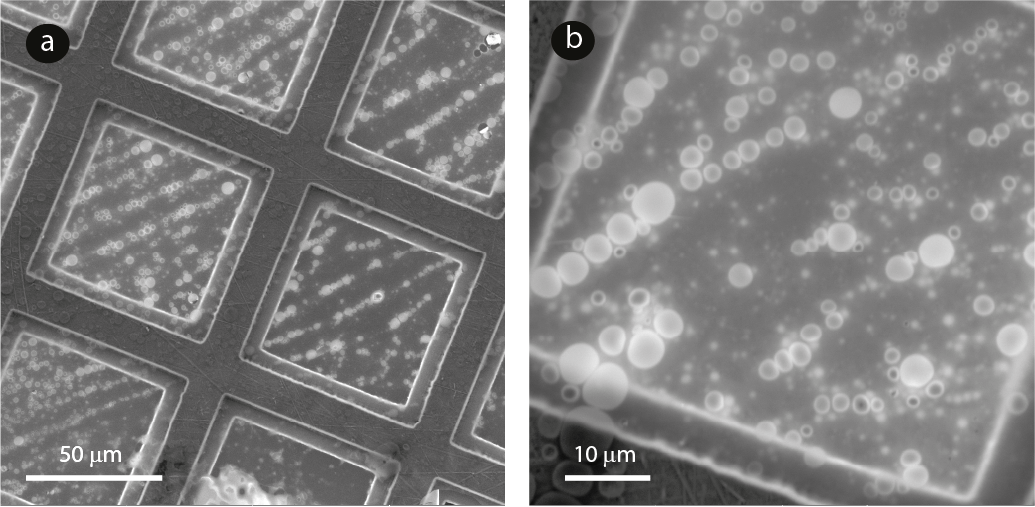


**Figure 9.** Microparticles contained within the dried pellet (TEM images). Note that the roundish microparticles are found all over the grid and are the primary type of particles

.


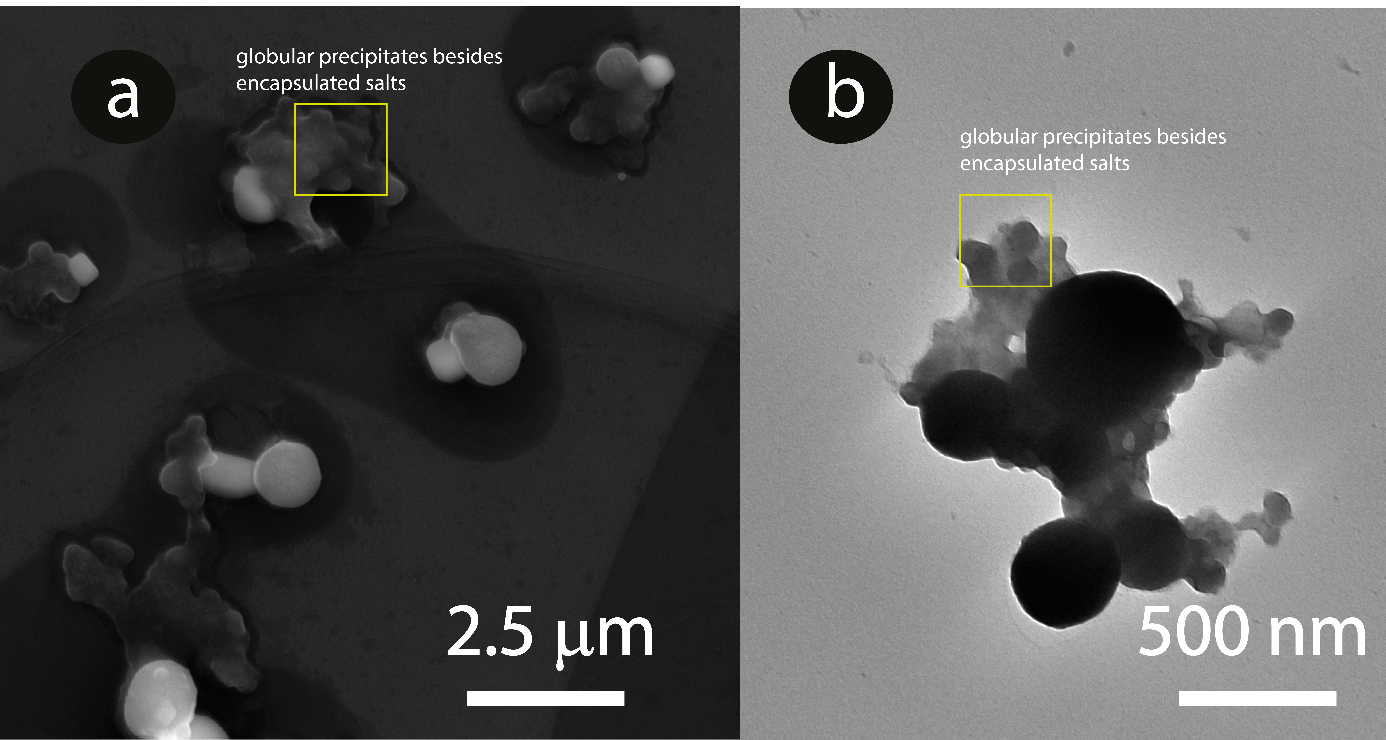


**Figure 10**. **a** SEM **b** TEM micrographs of the particles precipitated at 10000 rpm.

**
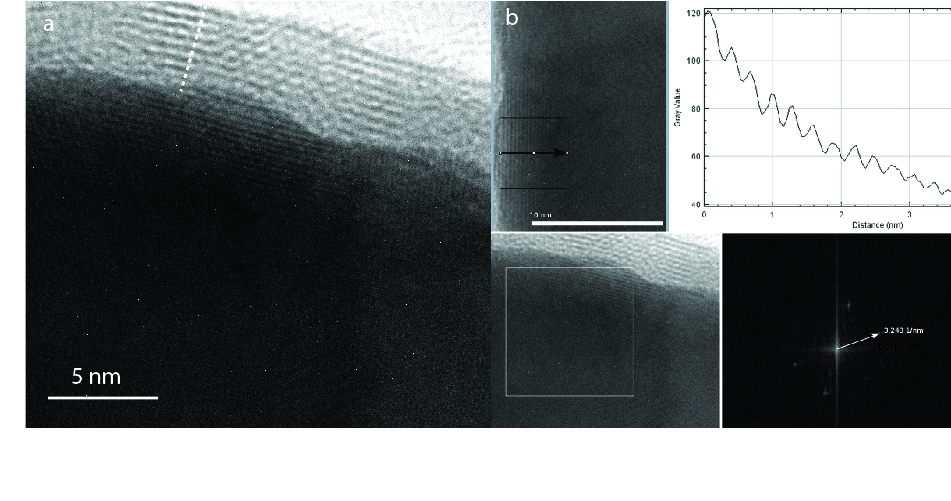
Figure 11.** High-resolution TEM image of crystalline particle surrounded by an electron-transparent nm-thick coating. **a** The highlighted are with dashed dots shows a pitch of 4.5 Å. **b** Electron-dense crystalline area showing a d-spacing of 3.08 Å; analyzed using image J.


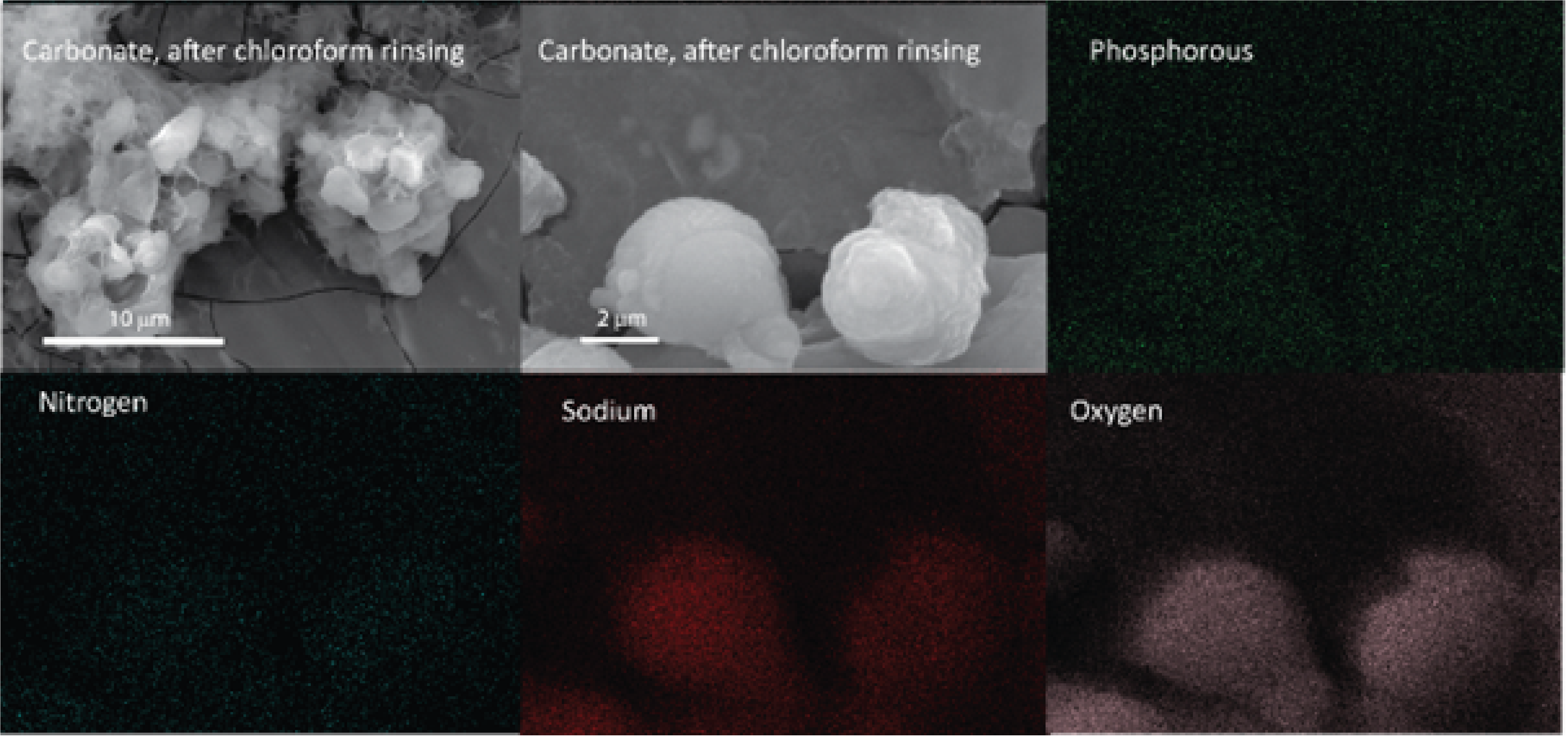


**Figure 12.** Microparticles contained within the pellet (SEM images) after being rinsed with chloroform and ethanol.


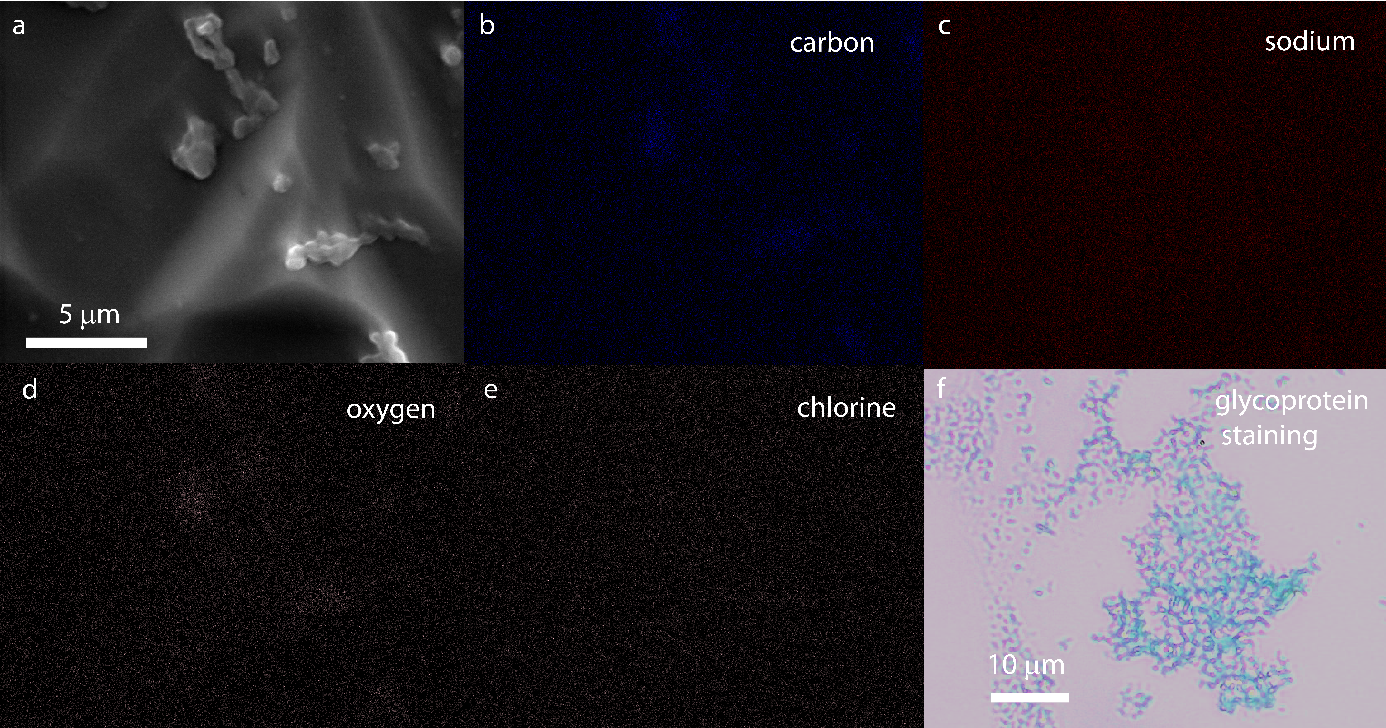


**Figure 13**. **a-e**. SEM-EDX mapping of the vesicles after dispersion in water and applying vortex. **f** Glycoprotein staining of the shells left behind after rinsing the microparticles with water. Glycosylated proteins could be part of the shells chemical composition.


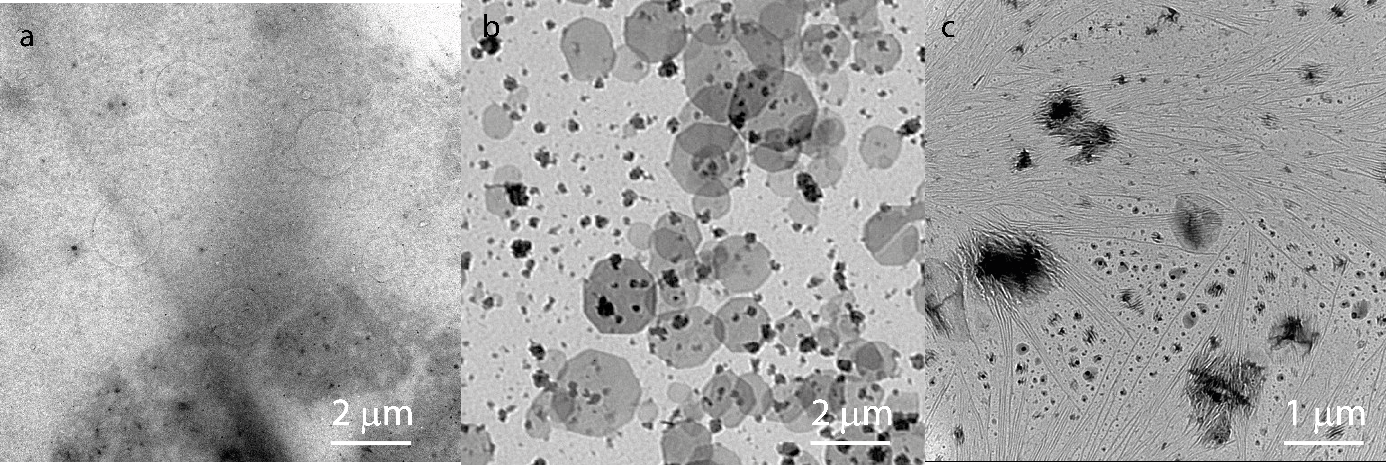


**Figure 14**. TEM micrographs of **a.** Slime supernatant film before staining. Notice that no electron-dense particles are imaged, and that roundish translucent structures are observed. The

TEM grids containing the absorbed supernatant were stained with a solution **b** 0.1 wt.% osmium tetroxide in PBS and of 0.4 wt.% uranyl acetate in PBS, **c** 0.4 wt.% uranyl acetate in PBS. Note that the translucent roundish structures may be associated with lipids.


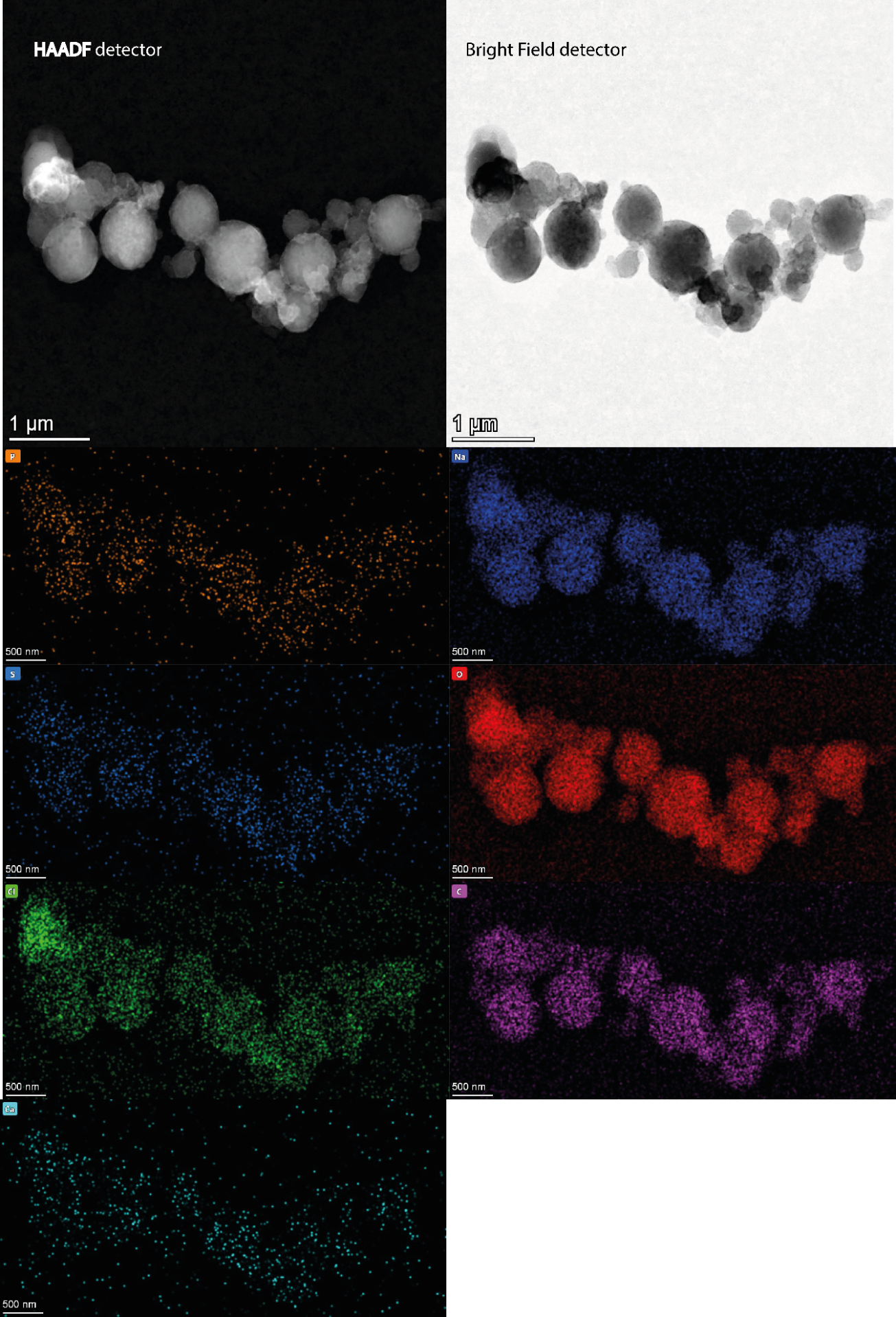
**Figure 15.** STEM-EDX analysis of the elemental composition of the rounded-edge particles shown before chloroform and ethanol rinsing. Note the other elements composed the particles beside the C, Na, O but in much lower amount ( Ca, Cl, P, K, S)


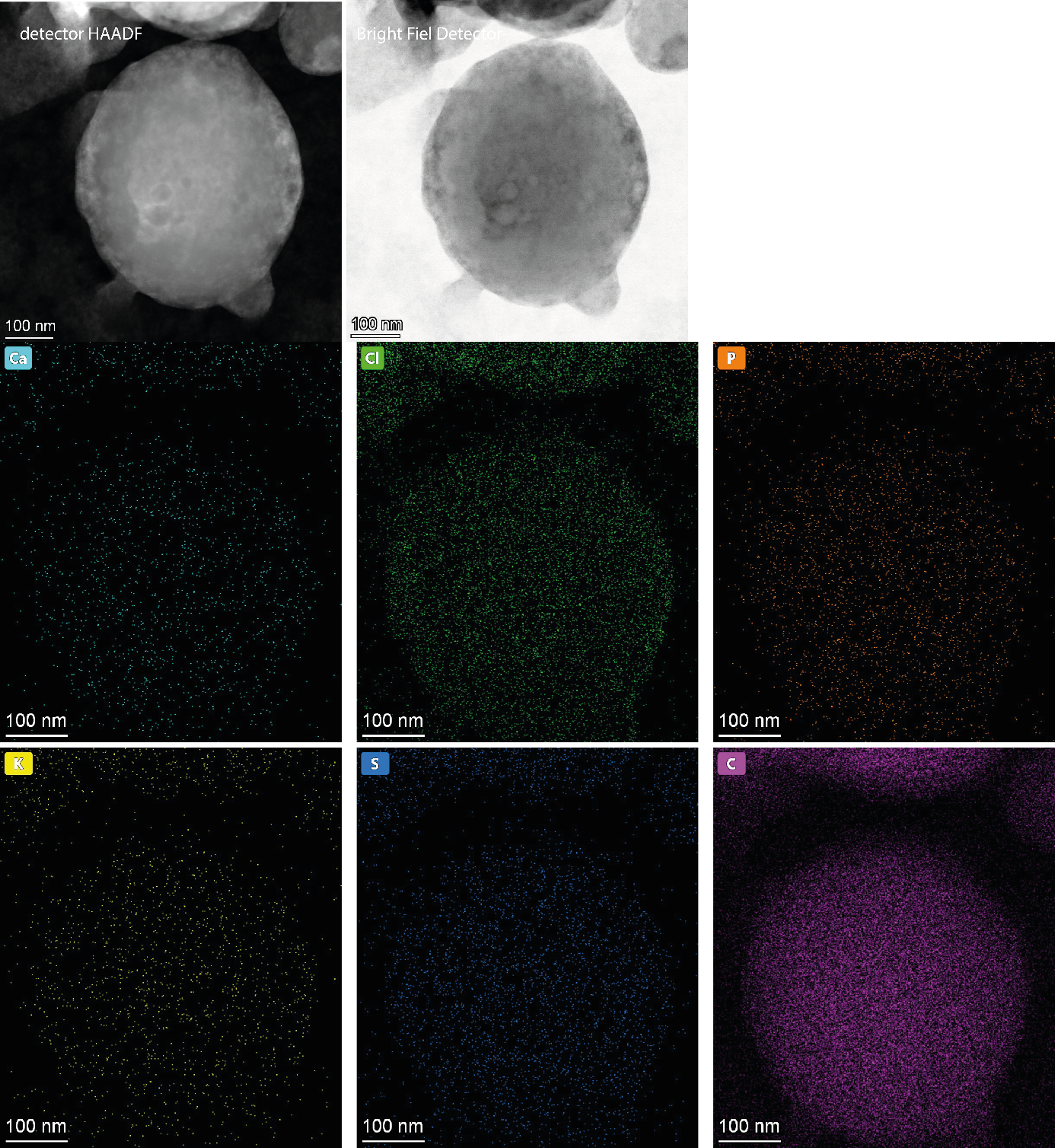


**Figure 16.** Complementary mapping of the elemental composition of the rounded-edge particles shown in Figure 5. Note the other elements composed the particles beside the C, Na, O but in much lower amount ( Ca, Cl, P, K, S) .

**
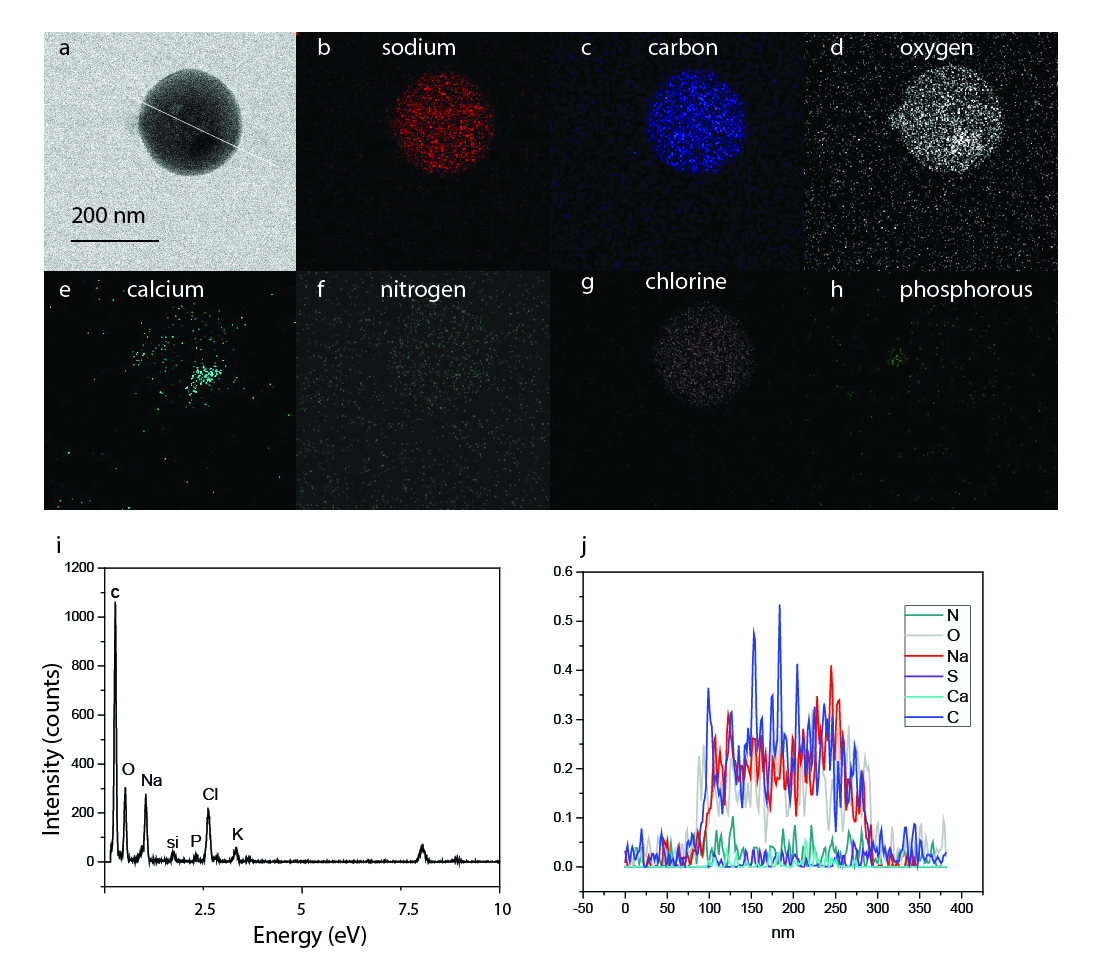
**

**Figure 17**. Elemental composition of the rounded-edge particles contained within the liquid expelled dried slime. STEM-EDS elemental maps of a single roundish-particle confirmed the low quantity of nitrogen and high carbon concentration. Lower amount of nitrogen and other elements are expected as part of the elemental composition as they are dispersed in a protein-based slime these particles might be biomineralized by proteins.

**Table 2**. Elemental composition of the unexpelled dried slime, encapsulated salts aggregates, and single particles isolated from liquid unexpelled slime, as determined by EDS. The values presented are the average of 30 independent particles. Measurements performed at 18 kv and 15 eV.

| **Element** | **Internal area**  **(Fig 2),** | **Encapsulated**  **salts aggregates**  **Fig. 1 f** | **Encapsulatedsalts aggregates**  **Fig. 1 f**  **at%** | **Sodium carbonate**  **Derivative**  **(rounded-edge particles)** | **Sodium phosphate**  **derivative** | **Sodium silicates** |
| --- | --- | --- | --- | --- | --- | --- |
|  |  |  |  |  |  | **derivative** |
|  | **Slime unexpelled** |  |  |  |  | **Na_2_O(SiO_2_)_x_** |
|  |  |  |  | **NaC_x_O_m_*(H2O)_n_** | **Na_2_HPO_4_*(H2O)_n_** |  |
|  | **at%**  **(area highlighted in Fig. 2c)** | **at%** |  | **at%** | **at%** | **at%** |
| **N** | 4.5±0.6 | 2.1±0.6 | 3.6±0.6 | 2.31±1.2 | 1.7±0.9 | 2.1±0.5 |
| **C** | 10.9±3 | **15.7±7** | **60±8.8** | **19.8±2.2** | 1.2±0.8 | 3.9±0.8 |
| **O** | 30.9±6 | **38.2±9** | **2±0.4** | **59.6±7.3** | **37± 8.3** | **56.4±7.4** |
| **Na** | 27.6±7 | **19.7±3** | **20.5±5** | **17.±1.1** | **29.1± 5.3** | **11.4±3** |
| **P** | 4.1±0.5 | **13.9±1.3** | 0.2±0.1 | 0.1 | **19.2** | 4.6±2 |
| **S** | 2.8±0.7 | 1.2±0.4 | - | 0.1 | 1.3 | 0.1 |
| **Cl** | 4.6±0.3 | 3.3±0.9 | **13.5±4** | 0.6 | 2.38±0.9 | 2.2±0.1 |
| **Ca** | 4.2±0.9 | 1.2±0.3 | - | 0.1 | 1±0.4 | 2±0.3 |
| **K** | 1.9±0.8 | 2.3±0.5 | - | 0.1 | 6.2±2.9 | - |
| **Si** | 4±0.9 | 1.4±0.4 | 0.1 | - | - | **17.3±4.9** |
| **Mg** | 4.4±0.3 | - | - | - | - | - |


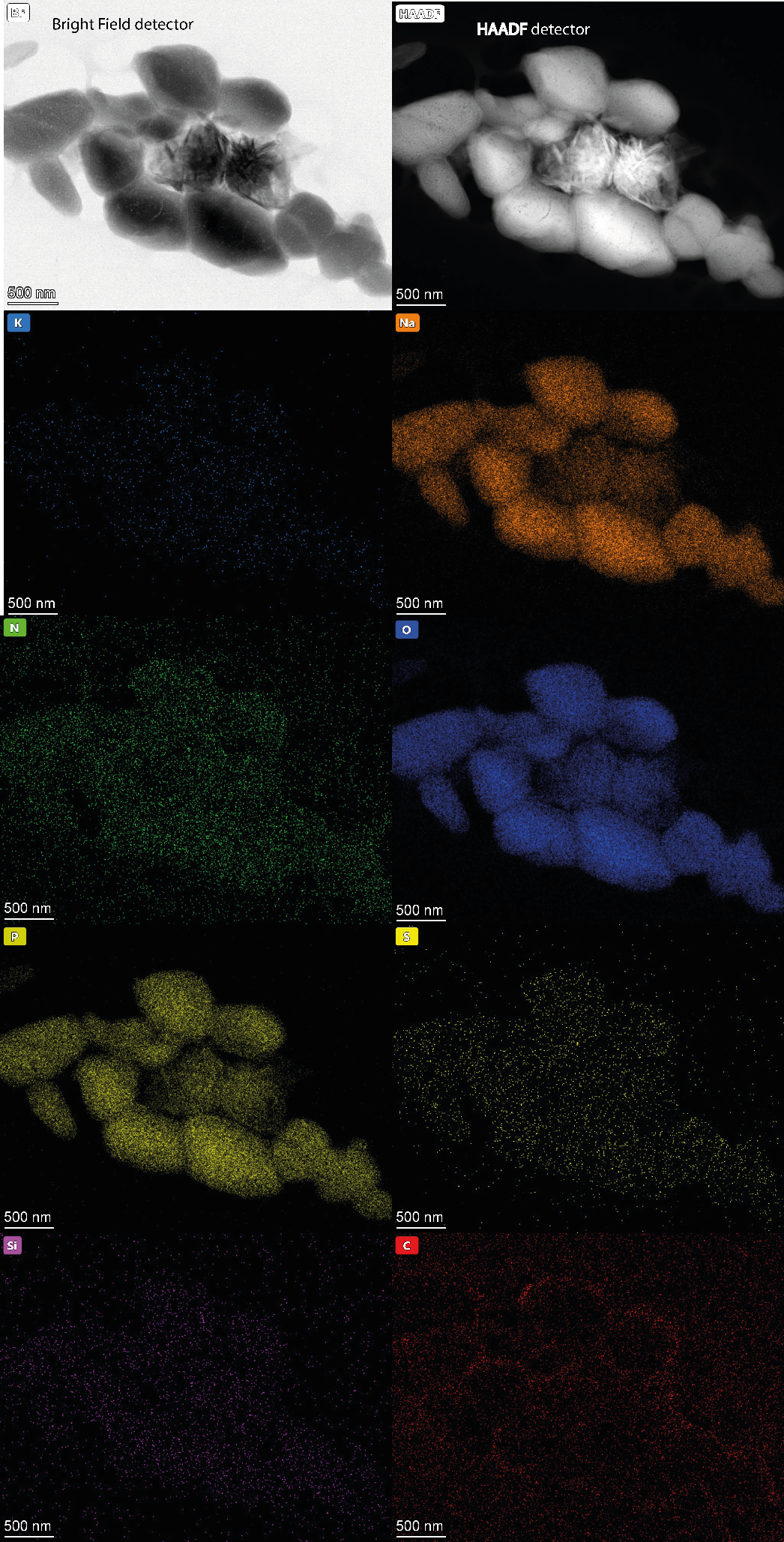


**Figure 18.** Complementary mapping of the elemental composition of the phosphate particles shown in Figure 6 from the main mansucript. Note the other elements composed the particles beside the P, Na, O but in much lower amount ( Ca, Cl, P, K, S)


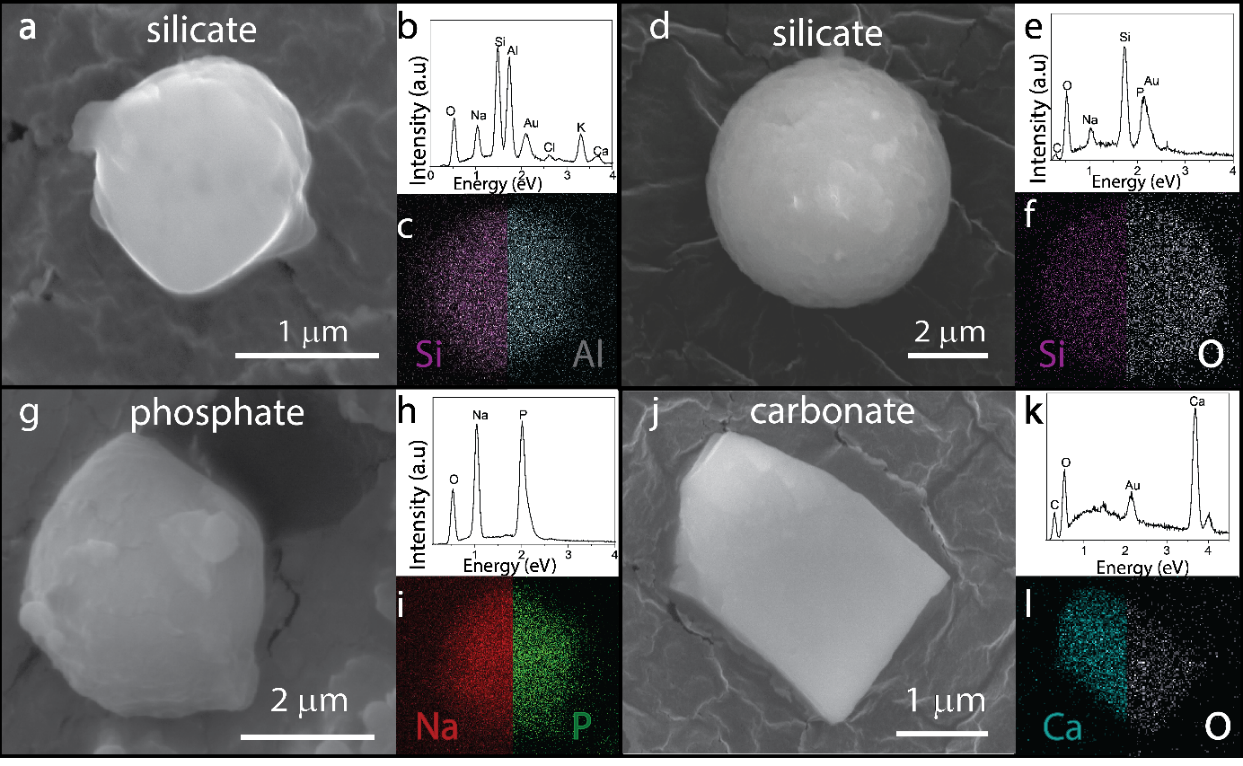


**Figure 19 .** Characterization of isolated particles. SEM image of **a** aluminum silicate **d** sodium silicate **g** sodium phosphate and **j** calcium carbonate representative isolated particles. **b**, **e, h** and **k** EDS spectra of a, d, g and j, respectively. EDS elemental map showing the two higher element with higher concentration detected: **c,f, i** and **j** of particle shown in a, d, g and j, respectively.

**Table 3**. Values of 2 theta calculated using the values of d-spacings calculated analyzing the TEM-SAED micrograph (120 KeV) shown in Fig. 4 d. Higher gray scale value correspond to a brighter pixel and is correlated to a certain distance in the reciprocal space. The TEM-SAED micrographs were analyzed using Image J.

**Table 3**. Values of 2 theta calculated using the values of d-spacings calculated analyzing the TEM-SAED micrograph (120 KeV) shown in Fig. 5 d. Higher gray scale value correspond to a brighter pixel and is correlated to a certain distance in the reciprocal space. The TEM-SAED micrographs were analyzed using Image J.

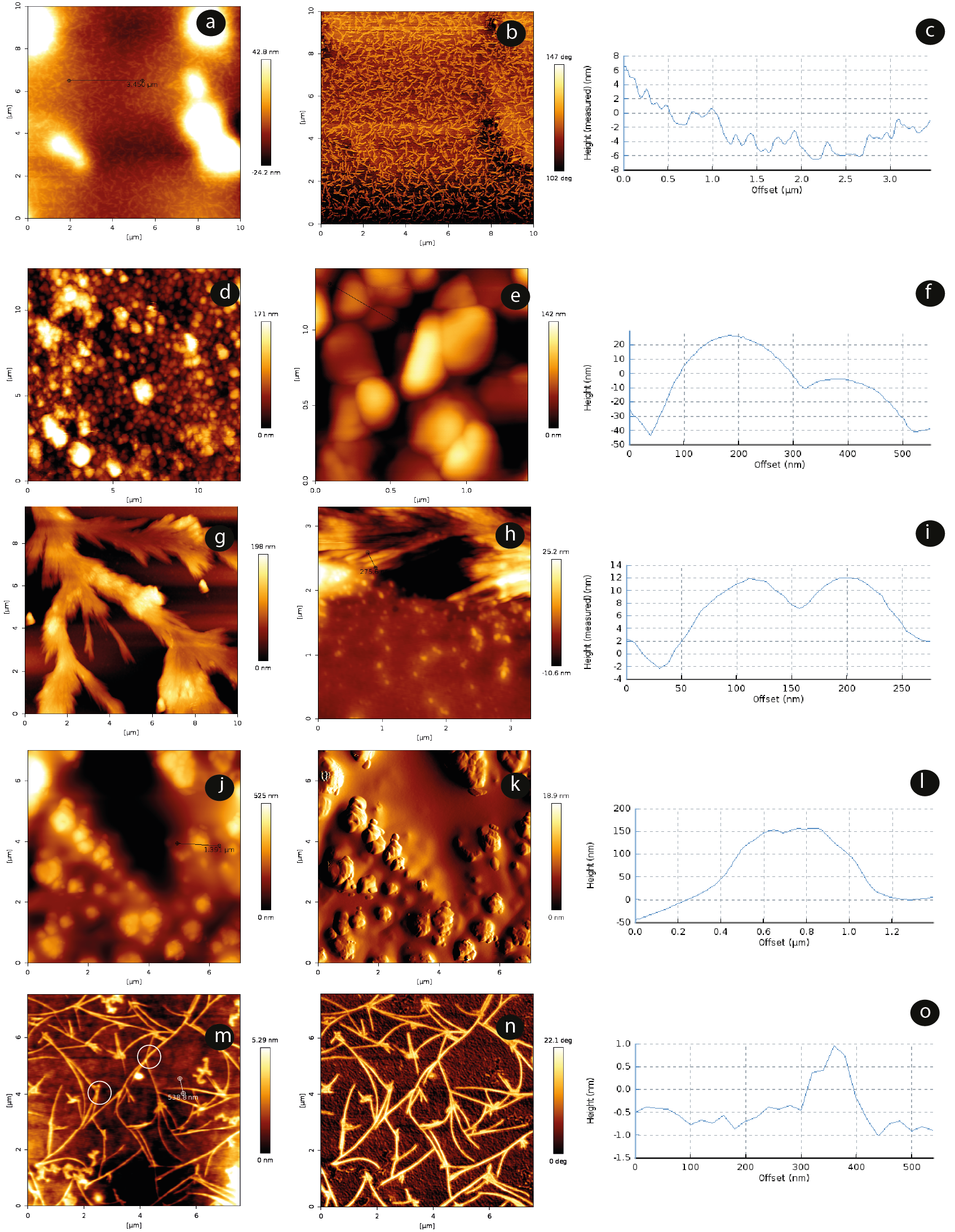


**Figure 20.** AFM height images of the slime **a** dialyzed, **d and e** a after adding NaCl at a 1.5 wt % concentration, **g and h** after adding NaCl at 1.5 wt % and sodium carbonate 1.5 wt% concentration, at a pH=7, **j** after adding NaCl at 1.5 wt % and sodium tripolyphosphate 1.5 wt% concentration, at a pH=7,  **m** after adding with NaCl 1.5 wt % and sodium tripolyphosphate 1.5 wt% concentration at a pH=9. **b** Phase image from a . **k** amplitude image from j. **n** Phase image from l **.c, f, I , l and o** Line cross-sections highlighted in a, d, g, j and m, respectively. The protein concentration was 20 mg/mL, approximately. NaOH and HCl 1 M was used to adjust the pH. A mica substrate freshly cleaved was used to deposit the solutions. 10 µL of each solution was let in dried at environmental conditions on the surface.

The proteins morphology dependence on the presence of NaCl, Na_2_CO_3_, and Na_5_P_3_0_10_ at pH=7 and pH=9 were studied using AFM. The salts wt% were kept at a similar range of the carbonic acid concentration contained in the native slime. The slime reconstituted in water and dialyzed showed individual fibers that formed small aggregates. Adding NaCl 1.5 wt% to the dialyzed slime catalyzed the formation of similar nanoglobules found in the native slime (slime/NaCl), Fig. 19 d-f. The average height of the nanoglobules was 110 ± 33 nm. Longer fibers ( several micrometers long) that agglomerate in the y and x directions were formed when Na_2_CO_3_ was added to the slime/ NaCl, Fig 19 g to h. Adding Na_5_P_3_0_10_ to the dialyzed slime/NaCl drove the aggregation of the nanoglobules in particles with heights of 340 ±87 nm, Fig. 19 j-l. The increment of the pH of this sample caused the formation of micrometer long and approximately 200 nm diameter fibers that were interacting mainly at the terminal areas. The results corroborate the type of salt and pH dependence on the protein morphology and interactions.

.
